# Global identification of *Chromobacterium violaceum* T6SS effectors reveals an Rhs antibacterial toxin featuring FIX and ADP-ribosyltransferase domains

**DOI:** 10.1016/j.jbc.2026.113216

**Published:** 2026-06-01

**Authors:** Júlia A. Alves, Genady Pankov, Andrew M. Frey, Matthias Trost, Germán G. Sgro, Sarah J. Coulthurst, José F. da Silva Neto

**Affiliations:** 1 Departamento de Biologia Celular e Molecular e Bioagentes Patogênicos, Faculdade de Medicina de Ribeirão Preto, Universidade de São Paulo, Ribeirão Preto, São Paulo, Brazil; 2 School of Life Sciences, University of Dundee, Dundee, UK; 3 Biosciences Institute, Newcastle University, Newcastle-upon-Tyne, UK; 4 Departamento de Ciências BioMoleculares, Faculdade de Ciências Farmacêuticas de Ribeirão Preto, Universidade de São Paulo, Ribeirão Preto, São Paulo, Brazil

**Keywords:** type VI secretion system, bacterial toxin, bacterial competition, secretome, mass spectrometry, bacterial genetics, ADP-ribosylation, protein secretion

## Abstract

Bacteria coexist in polymicrobial communities where they engage in complex interactions, including interbacterial antagonism. The environmental bacterial pathogen *Chromobacterium violaceum* possesses an active type VI secretion system (T6SS), which relies mainly on VgrG3 for its activity and role in interbacterial competition. However, the arsenal of toxic effectors delivered by this T6SS remains unknown. Here, we identify the repertoire of *C. violaceum* T6SS effectors and characterize a novel antibacterial Rhs-family effector, RhsF (Rhs with a FIX domain), and its cognate immunity protein, RhsFi. Using mass spectrometry analyses of secreted proteins and proteins co-immunoprecipitated with VgrG3, we identified six novel effector candidates, namely four phospholipases, a protein of unknown function, and the previously uncharacterized Rhs protein, RhsF (CV_1431). RhsF contains an N-terminal FIX domain and can intoxicate susceptible bacteria in a T6SS-dependent manner. The action of the C-terminal toxin domain of RhsF (RhsF-CT) is prevented by RhsFi (CV_1430), confirming that RhsF-RhsFi comprises an effector-immunity pair. The structure of the RhsF-CT/RhsFi complex determined by X-ray crystallography (1.85 Å resolution) revealed that RhsF-CT shares structural similarity with ADP-ribosyl transferase toxins and that RhsFi inhibits toxicity *via* direct occlusion of the RhsF-CT catalytic site. Functional assays indicated that RhsF-CT ADP-ribosylates RNA *in vitro* and that RhsF toxicity requires a catalytic triad composed of R1403, Y1456, and E1497 residues. Overall, our findings reveal effectors secreted by the T6SS of *C. violaceum*, establish RhsF as a potent antibacterial toxin, and confirm T6SS-dependent delivery of a FIX-containing Rhs protein, expanding the known repertoire of bacterial arms involved in microbial competition.

Bacteria live in polymicrobial communities where a variety of complex ecological interactions take place, including interbacterial competition. During interbacterial competition, bacteria compete for nutrients (exploitation competition) or use diffusible molecules and toxins delivered by specialized secretion systems to inhibit or kill competitors (interference competition) (1, 2). The Type VI Secretion System (T6SS) is a contractile nanomachine which delivers toxic effector proteins directly into neighboring cells and is employed by many Gram-negative bacteria to deliver antibacterial effectors as a means of interbacterial competition (3, 4, 5). There are four T6SS subtypes (T6SS^i^ to T6SS^iv^), with the canonical T6SS^i^ found in Proteobacteria. This T6SS is composed of three subcomplexes: a membrane complex (TssJLM), a baseplate (TssEFGK), and a bacteriophage tail-like structure consisting of a contractile sheath (TssBC) assembled around an inner tube (Hcp) which is tipped by a cell-puncturing spike (VgrG-PAAR). So-called “firing” of the T6SS involves contraction of the TssBC sheath, which propels the Hcp-VgrG-PAAR puncturing structure out of the secreting cell and into a recipient cell. The Hcp-VgrG-PAAR puncturing structure is decorated with effector proteins, thereby translocating them into recipient/target cells (6, 7, 8). Anti-bacterial effectors cause death or inhibition of growth of target bacterial cells unless they possess a cognate immunity protein. Effectors may be C-terminal toxin domains fused to the structural components VgrG, PAAR or Hcp (specialized effectors), or separate proteins that associate with VgrG, PAAR or Hcp non-covalently, sometimes *via* adaptor proteins (cargo effectors). Numerous T6SS effectors from distinct bacteria have been identified that act against competitor bacteria, by degrading the cell wall (amidases and glycosidases), the inner membrane (phospholipases), the nucleic acids (nucleases), or acting against other cellular targets (3, 9, 10). However, the repertoire of T6SS effectors remains to be experimentally determined for most bacterial species.

Rearrangement hot spot (Rhs) proteins are polymorphic toxins with a modular tripartite architecture (11, 12) and belong to the wider family of tyrosine/aspartate repeat-rich (YD-repeat) proteins found in bacteria, archaea, and eukaryotes (13, 14, 15). The N-terminal domain of Rhs proteins dictates their secretion pathway. In the case of T6SS-dependent secretion, most Rhs are specialized effectors containing an N-terminal PAAR domain (16, 17, 18) or, less commonly, are fused with VgrG proteins (19). One exception is TseI from *Aeromonas dhakensis*, an Rhs with an N-terminal domain of unknown function (20). The central core region of Rhs proteins is rich in tyrosine/aspartate (YD) repeats, which form a cocoon-like structure that encases the highly variable C-terminal toxin (effector) domain, delimited by a conserved PxxxxDPxGL motif (17, 18, 20). Biochemical and structural studies indicate that the toxic activity of the C-terminal domain depends on its release from within the Rhs shell, where it is otherwise protected (21, 17, 22, 23, 20, 24). For instance, in *Photorhabdus laumondii*, Rhs1 contains N- and C-terminal plugs that seal the toxic Tre23 domain within the hollow barrel of the core shell (23). In *Vibrio parahaemolyticus* RhsP, autoproteolysis induces significant structural rearrangements that facilitate β-barrel opening and RhsP dimerization (24). Like other T6SS effectors, the toxicity of Rhs proteins is known to be neutralized by their cognate immunity proteins (11, 10, 20, 24, 25), yet structural information on Rhs effector-immunity complexes remains limited.

*Chromobacterium violaceum* is a Gram-negative β-proteobacterium that inhabits soil and water in tropical and subtropical regions and causes rare but deadly infections in humans and other animals (26, 27, 28). *C. violaceum* kills Gram-positive bacteria by delivering the purple antibiotic violacein in outer membrane vesicles (29, 30). Recently, we demonstrated that *C. violaceum* strain ATCC 12472 can outcompete Gram-negative bacteria using a single quorum-sensing-regulated T6SS (31) and several potential effectors encoded by genes located in the main T6SS gene cluster and four ‘orphan’ VgrG-associated gene clusters were identified. Among the six VgrG proteins, VgrG3 appeared to play the most prominent role in the activity and function of the *C. violaceum* T6SS (31). Here, we aimed to identify the antibacterial effectors secreted by the T6SS of *C. violaceum* through mass spectrometry analyses of secreted proteins and proteins co-immunoprecipitated with VgrG3 from cellular fractions. Combining these two approaches, we identified several novel potential effectors and adaptor proteins, in addition to two new PAAR proteins that are not linked with other T6SS gene clusters. Among the candidate T6SS-delivered effectors, we performed genetic, biochemical, and structural characterization of a new Rhs effector-immunity pair, RhsF-RhsFi. Our data reveal that RhsF acts as an antibacterial effector whose C-terminal domain is an ADP-ribosyltransferase toxin and is inhibited by its cognate antitoxin, RhsFi. RhsF also represents the first experimentally confirmed example of a T6SS-dependent Rhs effector with a FIX-containing N-terminal delivery domain.

## Results

### Global identification of effectors secreted by the *C. violaceum* T6SS

**Figure 1:**
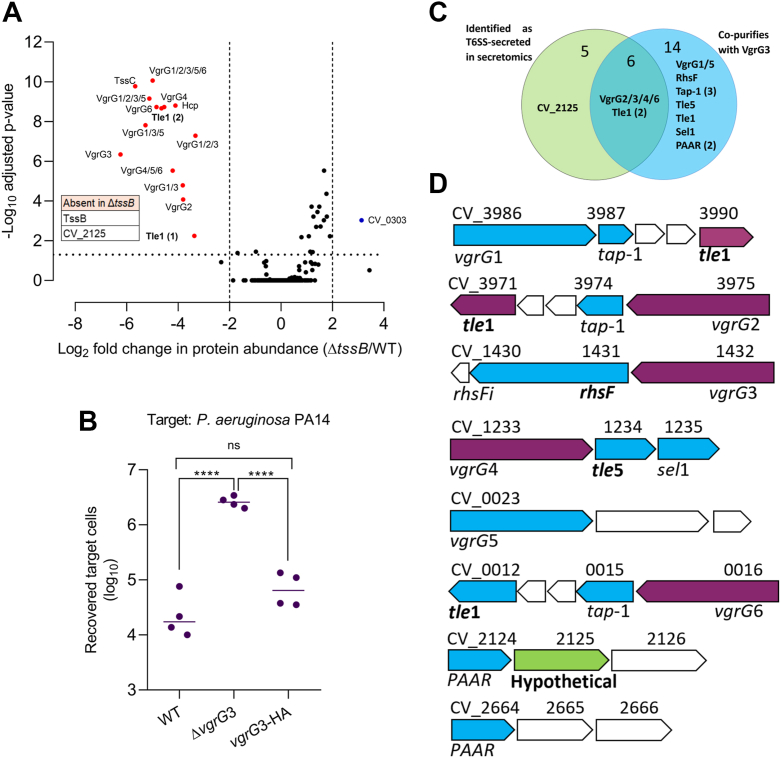
**Global identification of proteins secreted by the T6SS of *C. violaceum*.***A,* label-free quantitative mass spectrometry analysis of the secreted protein fraction obtained from *C. violaceum* grown in minimal medium. The volcano plot summarizes the comparison of proteins identified in the culture supernatant of wild-type *C. violaceum* ATCC 12472 (WT) and the Δ*tssB* mutant. Colored dots indicate proteins with significantly altered abundance in the Δ*tssB* mutant compared with the wild type (log2 fold change ≤ −2 or ≥ 2, *p*-value < 0.05, n = 7 independent biological replicates). Proteins CV_2125 and TssB were identified in the wild-type supernatant samples but were not detected in Δ*tssB* and therefore are not plotted. Further details for individual proteins are given in Table 1, Table 2. *B,* interspecies T6SS-dependent antibacterial activity. Recovery of *P. aeruginosa* PA14 target cells following co-culture with wild type *C. violaceum* or mutants carrying an in-frame deletion of *vgrG3* (Δ*vgrG3*) or encoding VgrG with a C-terminal HA tag at the normal chromosomal location (*vgrG*3-HA). Data are presented with a line showing the mean and individual data points overlaid (n = 4 biological replicates; ∗∗∗∗*p* < 0.0001, ns not significant; one-way ANOVA with Tukey’s test). *C,* Venn diagram showing the number of proteins identified by each strategy, with proteins of interest highlighted. *D*, genomic organization of genes encoding the identified T6SS-associated proteins. *Blue arrows*: proteins identified *via* VgrG3 co-immunoprecipitation (Co-IP); *Green arrows*: proteins identified *via* secretomics analysis; *Purple arrows*: proteins identified by both approaches. Genes encoding potential effectors are indicated in *bold*.

We previously demonstrated that the *C. violaceum* T6SS plays an important role in interbacterial competition and that VgrG3 is the most important among the 6 *C. violaceum* VgrG proteins for this T6SS-mediated killing (31). However, the T6SS-delivered antibacterial effectors remained to be identified. To identify effectors secreted by the *C. violaceum* T6SS, we employed two complementary strategies. The first involved using quantitative label-free mass spectrometry to compare the proteins in the secreted fraction (culture supernatant) of the wild type and the Δ*tssB* mutant (in which T6SS secretion is inactivated). This resulted in the identification of 10 non-redundant proteins significantly enriched, or exclusively present, in supernatant of the wild type strain, indicating that they are secreted in a T6SS-dependent manner (Figs. 1, *A*, *C* and S1*A*, Table 1). Of these, five proteins are secreted T6SS core components (Hcp, VgrG2, VgrG3, VgrG4, and VgrG6), two are phospholipases of the Tle1 family (CV_3990, associated with VgrG1, and CV_3971, associated with VgrG2), and one is a hypothetical protein (CV_2125), whose gene is organized in operon with a gene encoding a PAAR protein (Fig. 1*D*). The high similarity among the six VgrGs encoded by this bacterium, which share 71 to 93% amino acid identity, makes it challenging to identify specific VgrGs by mass spectrometry analysis. For that reason, some hits do not correspond to a specific VgrG (Fig. 1*A*, Table 1).

**Table 1:** T6SS-dependent proteins of *C. violaceum* identified by label-free quantitative mass spectrometry analysis of the secretome of the wild type and Δ*tssB* mutant

Accession	Identifier	Description	Log10 adjusted *p*-value	Log2 fold change Δ*tssB*/WT	Number of peptides
Q7NR06	CV_3979	TssB (T6SS sheath protein)	ND	ND	4
Q7NW65	CV_2125	Uncharacterized protein	ND	ND	5
Q7P238; Q7P245	CV_0016; CV_0023	VgrG5/6	ND	ND	3
Q7NY43	CV_1432	VgrG3	6.34	−6.24	6
Q7NR07	CV_3978	TssC (T6SS sheath protein)	9.775	−5.67	19
Q7NQZ9; Q7NY43; Q7P238	CV_0023; CV_1432; CV_3986	VgrG1/3/5	7.815	−5.26	3
Q7NQZ9; Q7NR10; Q7NY43; Q7P238	CV_0023; CV_1432; CV_3975; CV_3986	VgrG1/2/3/5	9.153	−5.12	4
Q7NQZ9; Q7NR10; Q7NY43; Q7P238; Q7P245	CV_0016; CV_0023; CV_1432; CV_3975; CV_3986	VgrG1/2/3/5/6	10.057	−4.99	6
Q7P245	CV_0016	VgrG6	8.727	−4.84	4
Q7NR10	CV_3975	VgrG2	4.078	−3.81	12
Q7NR14	CV_3971	Tle1 (phospholipase effector)	8.665	−4.65	33
Q7NYP0	CV_1233	VgrG4	8.727	−4.54	21
Q7NYP0; Q7P238; Q7P245	CV_0016; CV_0023; CV_1233	VgrG4/5/6	5.525	−4.21	5
Q7NQZ9; Q7NY43	CV_1432; CV_3986	VgrG1/3	4.784	−3.82	6
Q7NR08	CV_3977	Hcp	8.806	−4.1	29
Q7NQZ5	CV_3990	Tle1 (phospholipase effector)	2.241	−3.37	13
Q7NQZ9; Q7NR10; Q7NY43	CV_1432; CV_3975; CV_3986	VgrG1/2/3/5	7.286	−3.32	10
Q7P1B0	CV_0303	Probable indolepyruvate ferredoxin oxidoreductase	3.029	3.13	45

Proteins are included on the basis of the their abundance in the secreted fraction being > 4-fold altered in the Δ*tssB* mutant compared with the wild type (log2 fold change ≤ −2 or ≥ 2) with a significant *t* test (*p* < 0.05), or if they were not identified in the secreted fraction of Δ*tssB* but were detected in all replicates of the wild type. The table summarises the data from seven independent biological replicates of each strain. ND stands for not detected.

The second strategy used to identify new T6SS effectors was to identify proteins interacting with VgrG3 by co-immunoprecipitation from total cellular protein. For this purpose, a strain encoding a fusion of VgrG3 with a C-terminal haemagglutinin epitope tag (VgrG3-HA) at the normal chromosomal location was generated. The functionality of VgrG3-HA was confirmed by determining T6SS-dependent anti-bacterial activity against *Pseudomonas aeruginosa* in a co-culture assay, where the *vgrG3-HA* strain displayed similar activity to the wild type, in contrast with the Δ*vgrG3* mutant (Fig. 1*B*). Using quantitative mass spectrometry, 20 proteins were identified as being exclusively present, or significantly enriched, in the VgrG3-HA co-immunoprecipitation compared with the control (Table 2, Fig. S1, *B* and *C*). Of these, six proteins were also identified as being present in the secretome in a T6SS-dependent manner: the Tle1 phospholipases CV_3990 and CV_3971 and the VgrG proteins VgrG2, VgrG3, VgrG4, and VgrG6 (Fig. 1, *C* and *D*). An additional fourteen proteins were identified, including: VgrG1, VgrG5, another Tle1 family phospholipase (CV_0012), three Tap-1 adaptor proteins (CV_3987, CV_3974, and CV_0015), one Tle5 family phospholipase (CV_1234) and its putative immunity protein Sel-1 (CV_1235), one Rhs family protein (CV_1431), and two PAAR-containing proteins (CV_2124 and CV_2664) (Fig. 1, *C* and *D*, Table 2). Together, the two strategies revealed many candidate *C. violaceum* T6SS effectors, including four phospholipases, a protein of unknown function (CV_2125), and an uncharacterized Rhs protein (CV_1431).

**Table 2:** VgrG3-associated proteins identified by co-immunoprecipitation

Accession	Gene ID	Description	Mean LFQ intensity VgrG3-HA	Mean LFQ intensity WT	Peptides	Unique peptides	Sequence coverage [%]	-Log10 p-val VgrG3-HA/WT	Log2 FC VgrG3-HA/WT
Q7NQZ9	CV_3986	VgrG1	31.23	ND	102	11	89.7	ND	ND
Q7NR11	CV_3974	DUF4123 domain-containing protein Tap-1	31.24	ND	25	25	79	ND	ND
Q7NTA8	CV_3151	Uncharacterized protein	27.47	ND	17	17	45.3	ND	ND
Q7NUN3	CV_2664	PAAR domain-containing protein	26.95	ND	9	9	62.6	ND	ND
Q7NW66	CV_2124	PAAR domain-containing protein	29.04	ND	14	14	93.3	ND	ND
Q7NYN8	CV_1235	Lipoprotein Sel-1	31.34	ND	33	33	72.1	ND	ND
Q7NZ98	CV_1024	Flagellar biosynthesis protein FlhF	27.27	ND	16	16	57.2	ND	ND
Q7P246	CV_0015	DUF4123 domain-containing protein Tap-1	29.84	ND	21	21	64.5	ND	ND
Q7NQZ8	CV_3987	DUF4123 domain-containing protein Tap-1	31.66	24.90	25	25	85.9	ND	6.76
Q7P245	CV_0016	VgrG6	34.07	26.67	95	41	90.8	ND	7.40
Q7P238	CV_0023	VgrG5	33.20	22.74	101	17	88.2	4.93	10.46
Q7NR14	CV_3971	Tle1 phospholipase	32.71	23.14	67	67	76	4.35	9.57
Q7NY44	CV_1431	RhsF	36.69	27.54	241	241	95.8	9.75	9.15
Q7NQZ5	CV_3990	Tle1 phospholipase	32.38	24.10	44	44	60.8	3.63	8.28
Q7NR10	CV_3975	VgrG2	32.87	25.53	97	38	89.6	2.40	7.34
Q7P249	CV_0012	Tle1 phospholipase	30.78	23.47	38	38	71.5	7.30	7.31
Q7NYN9	CV_1234	Tle5 phospholipase	31.62	24.33	54	54	86.3	5.33	7.29
Q7NYP0	CV_1233	VgrG4	35.67	28.54	121	109	92.7	4.82	7.13
Q7NY43	CV_1432	VgrG3	36.56	29.56	129	38	92.2	6.06	7.01
Q7NYC6	CV_1348	Threonine--tRNA ligase. ThrS	26.97	24.72	14	14	33.5	2.39	2.25

Only proteins identified in all three replicates of VgrG3-HA are included. Proteins identified in two or three replicates of the control (WT) were excluded if they had an LFQ intensity value < 25 or a log2 fold change (VgrG3-HA/WT) < 3. ND stands for not detected.

### RhsF is an antibacterial toxin delivered by the *C. violaceum* T6SS and neutralized by RhsFi

**Figure 2:**
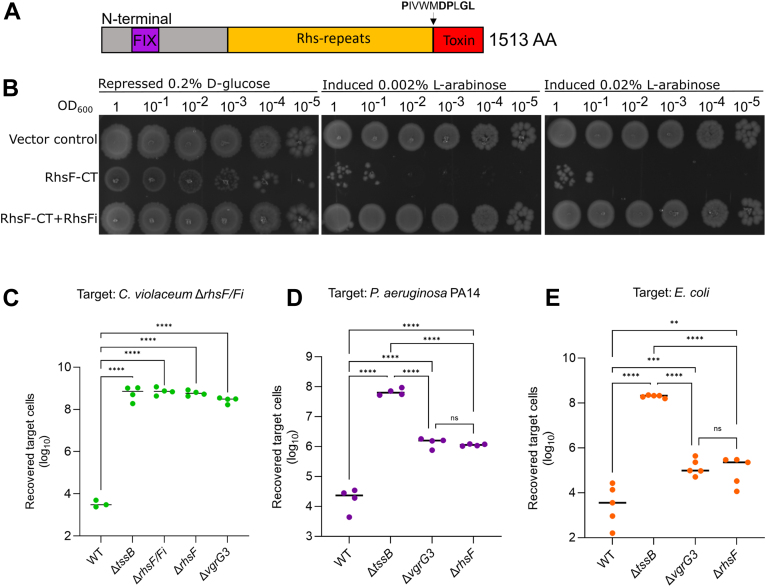
**RhsF is an antibacterial effector delivered by the T6SS.***A,* modular organization of RhsF highlighting the N-terminal FIX domain, the central Rhs repeat core, and the C-terminal toxic domain delimited by the conserved PxxxxDPxGL motif. *B,* growth of *E. coli* MG1655 carrying pBAD18-Kan (vector control) or derivatives directing the expression of the C-terminal toxin domain of RhsF (RhsF-CT) alone or co-expressed with RhsFi on LB media containing either d-glucose (repression) or l-arabinose (induction) for regulation of gene expression. *C,* intraspecies T6SS-dependent antibacterial activity. Recovery of *C. violaceum* Δ*rhsF/Fi* (target) following co-culture with wild type *C. violaceum* or mutants carrying in-frame deletions. *D-E*, interspecies T6SS-dependent antibacterial activity. Recovery of *P. aeruginosa* PA14 and *E. coli* following co-culture with wild type *C. violaceum* or mutants carrying in-frame deletions. *C–E*, Data are presented with a line showing the mean and individual data points overlaid (n ≥ 3 biological replicates; ∗∗∗∗, *p* < 0.0001; ∗∗∗, *p* < 0.001; ∗∗, *p* < 0.01; ns not significant; one-way ANOVA with Tukey’s *post hoc* test).

In the VgrG3 co-immunoprecipitation, we identified an Rhs family protein (CV_1431) that we named RhsF (for Rhs family protein containing a FIX domain). Analysis of this protein revealed a modular architecture typical of Rhs proteins (17, 18, 20). RhsF contains a FIX domain, which is associated with a number of T6SS substrates (32), in its N-terminal portion, a central Rhs core repeat region, and a C-terminal domain delimited by the PxxxxDPxGL motif (Fig. 2*A*). The *rhsF* gene is organized in a putative operon with *vgrG*3 (CV_1432) and a gene encoding a small hypothetical protein (CV_1430) that we named RhsFi (cognate immunity protein of RhsF) (Fig. 1*D*). To assess whether the C-terminal domain of RhsF (RhsF-CT) displays antibacterial toxicity, a gene encoding RhsF-CT (from after the predicted cleavage site delineated by the PxxxxDPxGL motif to the end of the protein, amino acids 1393–1513) was cloned under the control of an l-arabinose-inducible promoter, with and without the gene encoding the predicted immunity protein, RhsFi. Using these constructs to assess the impact of RhsF-CT expression revealed that the RhsF-CT is toxic when expressed in *Escherichia coli* and that this toxicity is neutralized by co-expression with RhsFi (Figs. 2*B* and S2), indicating that RhsF-RhsFi comprise an effector-immunity pair.

To determine whether the secretion of RhsF depends on the T6SS, we performed a co-culture assay for T6SS-dependent antibacterial activity. In this assay, a mutant strain of *C. violaceum* lacking the effector-immunity pair RhsF-RhsFi (in-frame deletion of *rhsF* and *rhsFi,* Δ*rhsF/Fi*) and marked with nalidixic acid resistance was used as the ‘target’ strain. Recovery of this RhsF-susceptible target strain was determined following co-culture with strains of *C. violaceum* with or without the ability to deliver RhsF. The number of colony-forming units of Δ*rhsF/Fi* recovered following competition against the *C. violaceum* wild-type strain was approximately 4 logs lower than following competition with strains with an inactive T6SS (Δ*tssB*) or lacking *rhsF* or *vgrG*3 (Fig. 2*C*), indicating that the delivery of RhsF depends on both the T6SS and VgrG3. The T6SS can secrete different effectors (toxins) in a single firing event, and this diversity ensures greater efficiency in killing competing bacteria (33). Given that we identified several proteins secreted by the *C. violaceum* T6SS that potentially act as antibacterial effectors (phospholipases from the Tle1 and Tle5 families, the hypothetical protein CV_2125, and RhsF), we evaluated the role of RhsF within this cocktail of secreted toxins. Interbacterial competition assays were conducted using *E. coli* and *P*. *aeruginosa* PA14 as targets. In both cases, deletion of *rhsF* significantly reduced the killing potential of *C. violaceum* compared to the wild-type strain (Fig. 2, *D* and *E*), indicating that RhsF plays an important role among the toxins secreted by the T6SS, through the toxic action of its CT and/or supporting delivery of other effectors as part of the secreted T6SS machinery.

### Structure of the RhsF-CT/RhsFi complex provides insights into toxin inhibition

To investigate the possible mechanism of action of the RhsF toxin, we initially used the RhsF-CT amino acid sequence to search for related proteins using protein Basic Local Alignment Search Tool (BLASTp) and for conserved motifs using the Motif Finder tool. Since no significant matches were retrieved, we attempted to determine the structure of RhsF-CT. His_6_-tagged RhsF-CT (amino acids 1393–1513 of RhsF) was co-produced with its immunity protein, RhsFi, and a stable complex of the two proteins was isolated by nickel-affinity chromatography (Fig. S3*A*). This complex was then subjected to size exclusion chromatography, where it eluted as a single peak with an estimated molecular weight of ∼29 kDa (Fig. S3*B*). Since RhsF-CT has a molecular weight of 14 kDa and RhsFi of 12 kDa, these data suggest the formation of a stable, heterodimeric RhsF-CT/RhsFi complex with a 1:1 stoichiometry. The complex was subjected to crystallization trials and following optimization of crystallization conditions, diffracting crystals were obtained (Fig. S3*C*).

**Figure 3:**
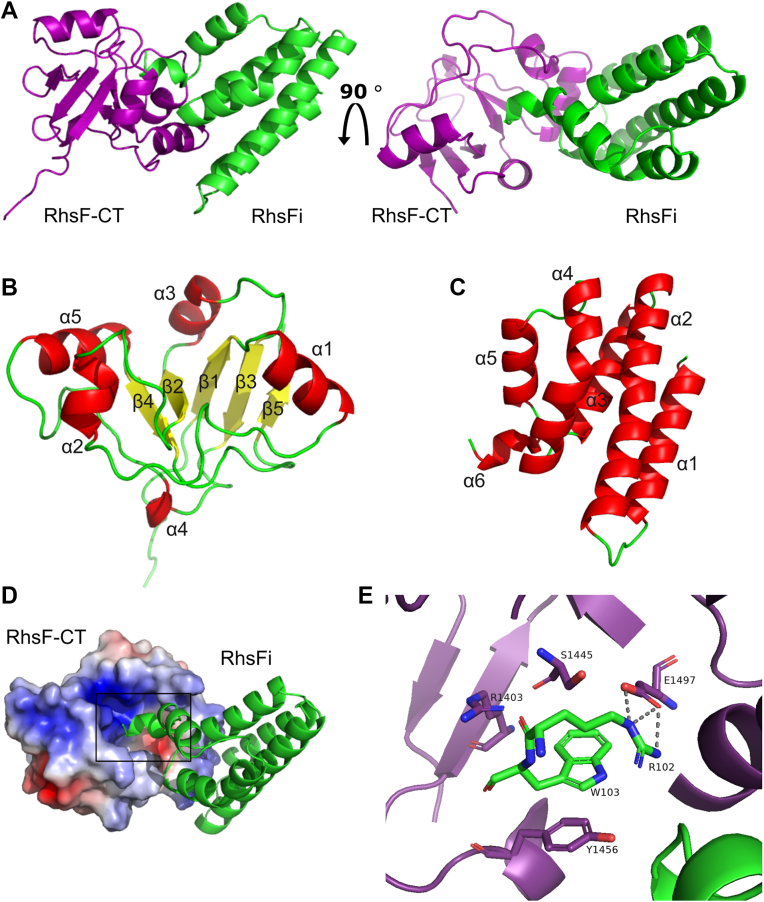
**Crystal structure of the RhsF-CT/RhsFi complex.***A,* cartoon representation of the RhsF-CT/RhsFi complex. RhsF-CT is shown in *purple* and RhsFi in *green*. *B–C,* cartoon representations of RhsF-CT (*B*) and RhsFi (*C*), with α-helices in red, β-strands in *yellow*, and loops in *green*. *D,* representation of the RhsF-CT/RhsFi complex showing the surface electrostatic potential of RhsF-CT, with positively charged regions in *blue* and negatively charged regions in *red*. RhsFi is shown in *green* cartoon. *Box highlight* interaction of RhsFi with putative catalytic site. *E*, close-up view of part of the interaction interface between RhsF-CT (toxin) and RhsFi (immunity protein). Key residues at the interface are highlighted: E1497, R1403, S1445, and Y1456 in the toxin (RhsF-CT, *purple*), and R102 and W103 in the antitoxin (RhsFi, *green*).

We determined the structure of RhsF-CT in complex with RhsFi to a resolution of 1.85 Å (Table 3; Fig. 3). The complex adopts a globular conformation in which RhsF-CT and RhsFi interact in a 1:1 stoichiometric ratio (Fig. 3*A*). The RhsF-CT has a structural core composed of a β-sheet divided into two units of antiparallel strands (β1, β3, β5, and β2, β4) interspersed with α-helices (Fig. 3*B*), whereas the immunity protein RhsFi is composed of six α-helices interspersed with small loops (Fig. 3*C*). The protein–protein interface covers approximately 1007.7 Å^2^, representing 13.5% of the solvent-accessible area of RhsF-CT (total area 7584.1 Å^2^) and 15.8% of RhsFi (total area 6300.1 Å^2^). The interaction is mediated by 28 residues of RhsF (22.2% of total residues) and 26 residues of RhsFi (25% of total residues), which form 12 hydrogen bonds and 15 salt bridges (Fig. S4).

**Table 3:** Data collection and structural refinement statistics

RhsF/RhsFi complex	
Data collection	
Space group	P1
Cell Dimensions	
a, b, c (Å)	34.83, 52.92, 66.39
α, β, γ (°)	72.03, 86.91, 71.84
Resolution range (Å)	47.85–1.85
*R*_merge_	0.087
*I/σI*	4.34 (1.85)
Completeness (%)	93.6 (47.85–1.85)
Redundancy	3.4
Refinement	
Resolution (Å)	1.85
No. reflections	34,223
*R*_work_/*R*_free_	0.1899/0.2194
No. atoms	7752
Protein	7314
Ligand/ion	0
Water	438
Average *B*-factors	29.82
Protein	29.14
Ligand/ion	0
Water	35.48
R.m.s. deviations	
Bond lenghts (Å)	0.007
Bond angles (°)	0.85

The RhsF-CT features an electropositive pocket, corresponding to its putative catalytic site, where four residues (R1403, S1445, Y1456, and E1497) interact closely with R102 and W103 in the α6 helix of RhsFi (Fig. 3, *D* and *E*). In this interface, R102 of RhsFi engages E1497 of RhsF-CT through an ionic bridge, and W103 directly interacts with RhsF residues S1445, Y1456, and R1403 *via* π–π and cation–π interactions. Immunity proteins associated with antibacterial effectors secreted by the T6SS typically function by occluding the catalytic site of the effector (34, 35). Therefore, the residues of RhsF-CT that interact with RhsFi may be key to understanding the function of this toxin.

### RhsF-CT exhibits structural features of ADP-ribosyltransferase toxins and modifies RNA *in vitro*

**Figure 4:**
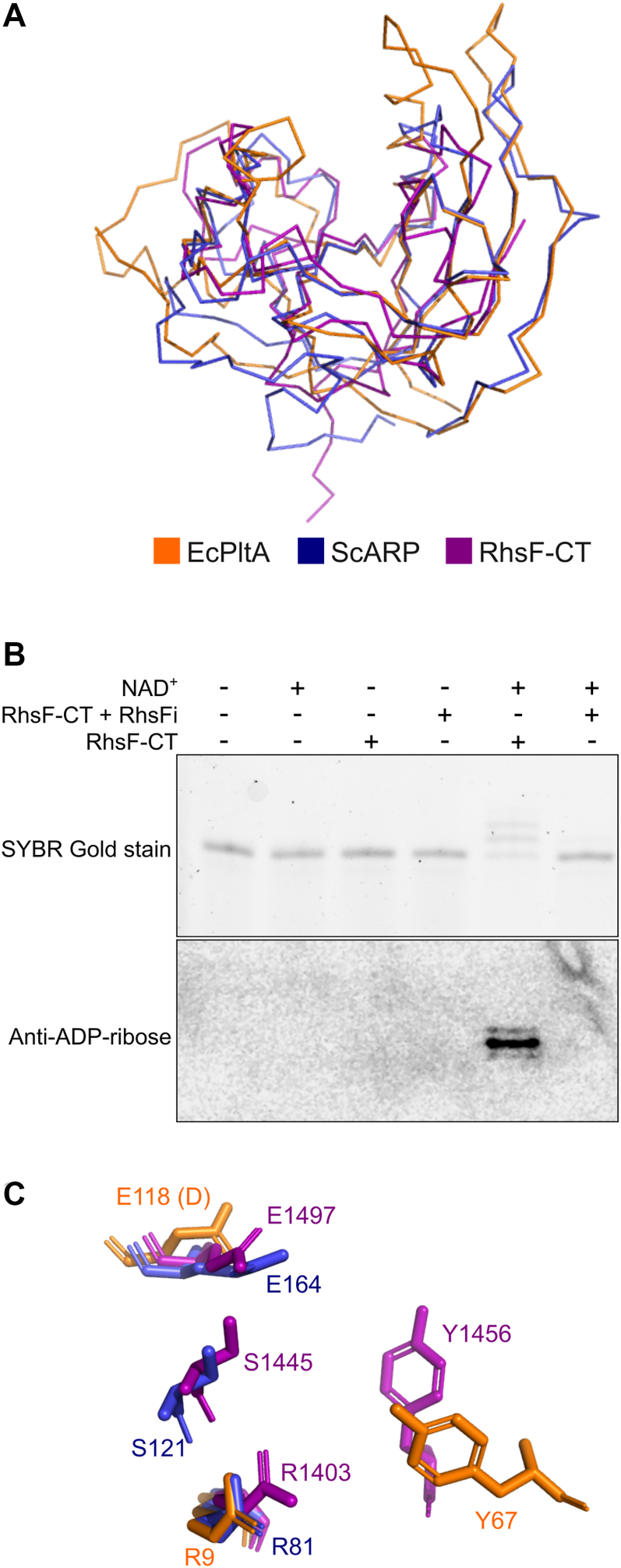
**RhsF-CT shares structural similarity with ADP-ribosyltransferase proteins and exhibits RNA ADP-ribosyltransferase activity *in vitro*.***A,* superimposition of RhsF-CT with ScARP (PDB 5zj4) and EcPltA (PDB 4z9d). Structures are depicted in ribbon representation, with EcPltA in *orange*, ScARP in *blue* and RhsF-CT in *purple*. *B, in vitro* assay to detect ART activity. Samples of purified RhsF-CT, or RhsF-CT and RhsFi, were incubated with an RNA oligonucleotide in the presence or absence of NAD^+^. RNA was separated using a polyacrylamide gel and either visualized directly by SYBR Gold staining, or ADP-ribosylated products were detected using an anti-poly/mono-ADP ribose antibody. *C,* close-up view of the relative positions of key residues from EcPltA and ScARP, along with their counterparts in RhsF-CT, based on the structural alignment in *A*. EcPltA residue E118 is shown as an aspartate because the available structure contains this substitution.

A search for proteins with structural similarity to RhsF-CT was conducted using PDB e-Fold (36). Three entries were retrieved using RMSD ≤ 2.5 and Z-score ≥ 5.0 as a threshold: the ScARP protein from *Streptomyces coelicolor*, bound and unbound with NADH (PDB 5zj5 and 5zj4, respectively), and the EcPltA protein from *E*. *coli* (PDB 4z9d) (Table S1). ScARP is an ADP-ribosyltransferase (ART) which ADP-ribosylates guanine nucleosides (37, 38). EcPltA is also an ADP-ribosyltransferase, encoded by certain pathogenic extraintestinal *E. coli* strains, which ADP-ribosylates mammalian inhibitory trimeric G-proteins, exhibiting cytotoxic activity (39). Bacterial ADP-ribosyltransferase toxins utilize NAD as a cofactor to catalyse the transfer of an ADP-ribose moiety onto an acceptor substrate, disrupting its function (40, 41). There is significant sequence divergence among bacterial ARTs, which can hinder their characterization based solely on sequence analysis. However, ARTs share a conserved core structure composed of a β-sheet formed by three antiparallel strands interspersed with α-helices (41), a feature that is also present in RhsF-CT (Fig. 3*B*). Structural superimposition of RhsF-CT with ScARP and EcPltA confirmed a shared structural similarity among these proteins (Fig. 4*A*).

To test experimentally whether RhsF-CT exhibits ART activity, we performed an ADP-ribosylation assay *in vitro* using a single-stranded RNA oligonucleotide as the substrate. This assay was chosen based on the observation that the catalytic site of RhsF-CT resides within an electropositive pocket (Fig. 3*D*), suggesting potential interaction with a negatively charged substrate such as nucleic acids. When incubated with purified RhsF-CT and NAD^+^, the mobility of the RNA was altered, and several bands were detected by polyacrylamide gel electrophoresis, implying modification of the RNA (Fig. 4*B*). Furthermore, these bands corresponding to modified RNA species cross-reacted with an anti-poly/mono-ADP ribose antibody, indicating that RhsF-CT can transfer ADP-ribose from NAD^+^ to the RNA. This ART activity of RhsF-CT was specific, since modification of RNA was prevented in the presence of RhsFi (Fig. 4*B*).

### The active site of RhsF-CT contains an atypical R-Y-E triad

The ART superfamily can be divided into two major groups depending on domain organization and conservation of active site motifs. Bacterial ARTs of class I, a group that includes diphtheria toxin, possess an active site composed of the H-Y-E motif, in which the nucleophilic histidine is proposed to bind to the nicotinamide group, orienting the NAD molecule in the catalytic pocket. The invariant glutamic acid residue is the key catalytic residue responsible for the coordination of NAD for hydrolysis before its binding to the substrate. ARTs of class II, on the other hand, present a triad composed of R-S-E motif, in which the nucleophilic arginine and serine residues positions and stabilizes the NAD molecule in the active site, and the highly conserved glutamic acid has a similar function as in class I ARTs (42, 40, 41). The catalytic residues in ScARP and EcPltA are R81-S121-E164 and R9-Y67-E118, respectively. We performed a structural superimposition to identify the equivalent residues in RhsF-CT. This indicated a high degree of spatial conservation with the arginine and glutamate residues from ScARP and EcPltA, which aligned with R1403 and E1497 in RhsF, respectively (Fig. 4*C*). The third component of the catalytic triad, however, could not be unambiguously identified by this method, as Y1456 in RhsF aligns with Y67 from EcPltA, while S1445 aligns with S121 from ScARP (Fig. 4*C*).

**Figure 5:**
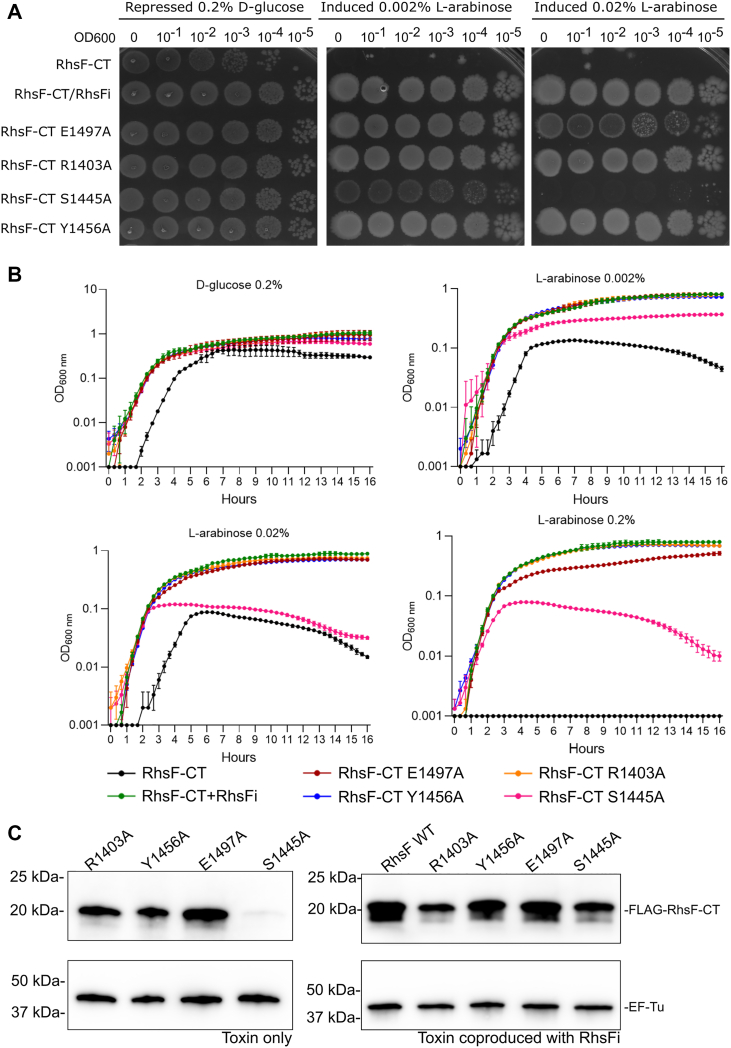
**Substitution of amino acids predicted to be important for enzymatic function of RhsF-CT results in a loss of toxicity.***A–B,* growth of *E. coli* MG1655 carrying pBAD18-Kan-based plasmids directing the expression of wild type RhsF-CT alone or co-expressed with RhsFi, or of RhsF-CT variants with the amino acid substitutions indicated on solid (*A*) or in liquid (*B*) LB media containing d-glucose or l-arabinose for repression or induction of gene expression, respectively. *C,* total protein samples from *E. coli* carrying plasmids directing the expression of RhsF-CT variants with an N-terminal 3xFLAG epitope tag (FLAG-RhsF-CT), with or without the immunity protein RhsFi, were subjected to immunoblotting to visualise levels of RhsF-CT in each case. Membranes were also probed using an anti-EF-Tu antibody to provide a loading control.

To verify the importance of the predicted catalytic triad residues of RhsF, the toxicity of variants of RhsF-CT carrying substitutions of these residues was tested by heterologous expression in *E. coli*. The growth of cultures of *E. coli* MG1655 carrying constructs directing the expression of RhsF-CT variants was assessed in both solid and liquid LB medium (Fig. 5, *A* and *B*). Individual substitution of residues E1497, R1403, and Y1456 with alanine resulted in loss of RhsF-CT toxicity in both conditions, whereas mutation of residue S1445 resulted in partial reduction of toxicity (Fig. 5, *A* and *B*). In some growth curves, a decrease in OD_600_ was observed for the toxic versions RhsF-CT WT and S1445A at later time points (Fig. 5*B*). However, this likely reflects a secondary consequence of intoxication rather than a lytic death mechanism, as no cell lysis was observed in *E. coli* expressing RhsF-CT during the time-lapse assays (Fig. S2). To ensure that the observed loss of toxicity was not due to compromised protein stability or expression, an N-terminal 3xFLAG tag was incorporated in each of the RhsF-CT variants to allow visualization of protein levels *via* immunoblotting. Upon co-expression with the immunity protein RhsFi, the levels of all RhsF-CT FLAG-tagged variants were similar to those of the wild-type FLAG-RhsF-CT (Fig. 5*C*). In the absence of the immunity protein, the non-toxic variants R1403A, Y1456A, and E1497A remained present at high levels, whereas a marked reduction in the levels of S1445A was observed, consistent with the partial loss of toxicity of this variant resulting from decreased protein stability (Fig. 5*C*). Toxicity assays performed with these FLAG-tagged variants (Fig. S5) mirrored the effects observed with the corresponding non-tagged versions (Fig. 5*A*). In the toxicity assays, a few colonies were observed at higher dilutions, suggesting the emergence of suppressors in the toxic versions of RhsF-CT (Figs. 5*A* and S5). Taken together, these results suggest that RhsF-CT is an ADP-ribosyltransferase with a catalytic triad most likely composed of residues R-Y-E.

## Discussion

In this study, we performed a global analysis of effectors secreted by the T6SS of *C*. *violaceum*. Combining two complementary mass spectrometry approaches, we identified six novel effector candidates, namely four phospholipases (CV_0012, CV_1234, CV_3971, and CV_3990), a hypothetical protein (CV_2125), and an uncharacterized Rhs protein (CV_1431). These findings reveal the broad repertoire of T6SS effectors employed by *C. violaceum* for interbacterial competition. Among these T6SS-associated effectors, we focused on an Rhs toxin-immunity pair, RhsF-RhsFi. We demonstrate that the FIX-containing RhsF protein is a potent T6SS-dependent antibacterial effector whose C-terminal toxin domain exhibits structural features of ADP-ribosyltransferase toxins, can ADP-ribosylate RNA *in vitro*, and can be neutralized by the immunity protein RhsFi *via* direct occlusion of its catalytic site.

Our combined proteomics analysis was effective in identifying: (i) effectors predicted previously *in silico* (Tle1 and Tle5 phospholipases and the Rhs protein CV_1431), which are encoded within the main T6SS cluster and the four orphan VgrG clusters (31, 43); (ii) a new potential cell-wall acting effector (CV_2125); (iii) Tap-1/TEC adaptor proteins associated with effector secretion (44, 45); (iv) two PAAR proteins not identified previously (CV_2124 and CV_2664), which are encoded outside of the *C. violaceum* T6SS clusters; and (v) all 6 *C. violaceum* VgrG proteins (Fig. 1, Tables 1 and 2). The CV_2124-CV_2126 and CV_2664-CV_2666 gene clusters likely form two operons encoding PAAR proteins (CV_2124 and CV_2664), putative cell-wall-acting effectors (CV_2125, which lacks predicted domains, and CV_2665, which contains a soluble lytic transglycosylase domain), and their putative signal peptide-containing immunity proteins (CV_2126 and CV_2666). We are currently investigating the function of these gene clusters. In our previous study, we showed that deletion of VgrG3, among all six VgrGs, has the greatest impact on T6SS firing and T6SS-mediated bacterial killing (31), but it was unclear whether all six VgrGs were expressed and functional. Here, our data provide evidence that all six VgrG proteins are either secreted and/or interact with VgrG3 (Tables 1 and 2), confirming that *C. violaceum* deploys all available VgrGs through its single T6SS. Since many cargo effectors are dependent on interaction with specific VgrGs for delivery (16, 46), encoding six VgrGs provides *C. violaceum* with a strategy to deliver a broad range of cargo effector toxins.

We also provide evidence that RhsF is a toxin delivered by the *C. violaceum* T6SS (Fig. 2). Rhs proteins secreted *via* the T6SS are typically specialized effectors that contain a PAAR domain at their N-terminus and/or are encoded alongside chaperones that mediate interaction with VgrG proteins; less commonly, they may also be fused to VgrG proteins (15, 26, 38, 42, 56, 23, 47). In contrast, we did not identify any chaperone genes adjacent to or within the *rhsF* operon, nor did we detect any candidate RhsF-associated chaperones in our mass spectrometry. Furthermore, the N-terminal region of RhsF lacks a PAAR domain and has instead a FIX (Found in type sIX) domain (Fig. 2). The FIX domain is found in the N-terminal or central regions of several predicted T6SS-secreted effectors and has been shown to be essential for the secretion, but not the toxicity, of a T6SS-dependent DNase effector from *V*. *parahaemolyticus* (32). Here, we show that an Rhs protein containing a FIX domain is secreted in a T6SS-dependent manner, providing experimental evidence that FIX can replace PAAR to direct secretion of Rhs-containing effectors by the T6SS. Our co-immunoprecipitation data suggest a direct interaction between RhsF and VgrG3, which contains a transthyretin-like (TTR-like) domain at its C-terminus. The TTR-like domain, or an Ig-like variant domain found in many VgrG proteins, has been implicated in specific interactions with the phospholipase Tle1 (48) or the Rhs effector Tse15 (22), respectively, allowing their secretion. These findings suggest the possibility of a conserved effector-VgrG interaction mechanism mediated by TTR-like or structurally related domains. Further investigations are required to determine whether the secretion of RhsF *via* the T6SS machinery involves direct interaction of its N-terminal FIX domain with the TTR-like domain of VgrG3.

Our atomic structure of the C-terminal toxic domain (RhsF-CT) of RhsF in complex with the immunity protein RhsFi revealed that RhsF-CT shares strong structural similarity with ADP-ribosyltransferases (ARTs) and provided structural insights into the mechanism of toxin inhibition *via* direct occlusion of its catalytic site by the antitoxin RhsFi (Figs. 3 and 4). Toxicity assays using variants of the RhsF-CT with single amino acid substitutions underscored the importance of three key residues: R1403, Y1456 and E1497 (Fig. 5). Bacterial ARTs are classified into two classes: class I, whose catalytic site consists of the H-Y-E triad, and class II, characterized by an R-S-E triad. In both cases, there is an ultra-conserved glutamate residue (E) essential for nearly all bacterial ARTs, and a nucleophilic histidine or arginine, typically preceded by a tyrosine residue (42, 40). Therefore, the RhsF-CT shares characteristics with ARTs of class I, but with an arginine instead of the classical histidine. Although ARTs are traditionally classified into these two main groups, a few bacterial ARTs with divergent catalytic motifs have been reported (25, 40, 49, 50), including the type III secretion system (T3SS) effector CteC, an ART of *C. violaceum* that ADP-ribosylates host ubiquitin during infection (51, 52). These atypical ARTs can diverge substantially from the canonical H-Y-E and R-S-E classes. For example, the CteC family adopts a D-E catalytic motif and operates through a distinct catalytic mechanism (51). The R-Y-E variation observed in RhsF-CT appears to be particularly unusual and has so far been reported only in the ART RhsP2 (25, 50). Nevertheless, structural and biochemical analyses of RhsP2 indicate that this non-canonical triad fulfills roles analogous to those of the catalytic residues in classical ARTs (50). Interestingly, RhsP2, like RhsF, ADP-ribosylates RNA (25), so it is possible that this triad is associated with activity against RNA.

The C-terminal toxic domains of Rhs proteins are very variable, including DNases, NADases, and ADP-ribosyltransferases (13, 18, 25, 47, 50, 53, 54). RhsF-CT displays catalytic and structural features of an ART toxin and exhibited ADP-ribosyltransferase activity *in vitro* by modifying an RNA probe (Fig. 4*B*), indicating it is likely to target one or more RNA species *in vivo*. However, further studies are required to demonstrate its precise molecular target *in vivo* and the mechanisms that trigger death or stasis during interbacterial competition. Although proteins are the most common substrates described for ART enzymes, some have been reported to modify nucleic acids, including RNA (18, 25, 37, 50).

Overall, we have characterized an important component of the antibacterial effector repertoire of the *C. violaceum* T6SS and demonstrated T6SS-mediated deployment of a FIX-containing Rhs protein. Our findings contribute to a deeper understanding of how bacteria utilize T6SSs and diverse antibacterial effectors to compete against rival bacteria and shape polymicrobial communities.

## Experimental procedures

### Bacterial strains, plasmids, and growth conditions

Bacterial strains and plasmids used in this study are listed in Table 4 and Table S2, respectively. *C. violaceum* and *E. coli* strains were cultured at 37 °C in Luria-Bertani (LB) (10 g/L tryptone, 5 g/L yeast extract, 10 g/L NaCl) liquid medium with shaking at 200 rpm or on solid LB medium (supplemented with 15 g/L agar). When necessary, antibiotics were added at the following concentrations: ampicillin 100 μg/ml, kanamycin 50 μg/ml, nalidixic acid 20 μg/ml, chloramphenicol 25 μg/ml, and streptomycin 100 μg/ml. To repress gene expression from pBAD18-Kan, 0.5% d-glucose was added to the media for cloning and maintenance. The minimal medium used for proteomic analysis of *C. violaceum* supernatant had the following composition: phosphate buffer (K_2_HPO_4_ 40 mM, KH_2_PO_4_ 8 mM), ammonium sulfate 0.1%, magnesium sulfate 0.41 mM, glucose 0.4%, Complete Supplement Mixture (CSM) Single Drop-Out: -Adenine (Formedium) 780 mg/l, and adenine 0.01%. Solid minimal medium was prepared by supplementing with 15 g/L agar. DNA cloning procedures in plasmids were performed using *E. coli* DH5α, except for constructs with the pKNG101 vector, which were generated using *E. coli* CC118λpir. The transfer of plasmid constructs into *C. violaceum* strains was carried out *via* triparental conjugation (*E. coli* CC118λpir along with *E. coli* HH26 (pNJ5000) as the helper strain).

**Table 4:** Bacterial strains used in this work

Bacterial strains	Description	Reference
*Escherichia coli*		
DH5α	Strain for cloning purposes	(55)
BL21 (DE3)	Protein overproduction strain. Chromosomal λDE3 encodes IPTG-inducible T7	Novagen
MG1655	K-12 strain. Used for toxicity assay	(56)
CC118λpir	Cloning host and donor strain for pKNG101-derived allelic exchange plasmids (λpir)	(57)
HH26 pNJ5000	Mobilizing strain for conjugal transfer	(58)
BW25113 *lacA*::kan	Keio collection strain with a mutation in the *lacA* gene. Used as a target strain for competition assays. KanR	Keio collection
*Chromobacterium violaceum*		
ATCC 12472	Wild type (Sequenced genome)	(27)
Δ*tssB*	ATCC 12472 with in frame deletion of *tssB* (CV_3979); this strain is also known previously as Δ*vipA.*	(31)
Δ*vgrG3*	ATCC 12472 with in frame deletion of *vgrG3* (CV_1432)	(31)
CV NAL	ATCC 12472 resistant to nalidixic acid due to a spontaneous mutation in the *gyrA* gene	(59)
JA01	CV NAL Δ*rhsF* (CV_1431) and Δ*rhsFi* (CV_1430)	This work
JA02 (Δ*rhsF*/Δ*rhsFi*)	ATCC 12472 with in-frame deletions Δ*rhsF* and Δ*rhsFi*	This work
JA03 (Δ*rhsF*)	ATCC 12472 Δ*rhsF*	This work
JA05 (*vgrG*3::HA)	ATCC 12472 encoding VgrG3 with a hemagglutinin (HA)-tag epitope at its C-terminus, at the normal chromosomal location	This work
*Pseudomonas aeruginosa*		
*P. aeruginosa* PA14 P_rpsg_-GFP	*P. aeruginosa* PA14. Used for the competition assay	(60)

### Construction of *C. violaceum* mutant strains

Null mutant strains of *C. violaceum* (non-polar in-frame deletion of a given gene) were generated by allelic exchange through homologous recombination (29, 31, 59) using the suicide vector pKNG101 (61, 62). The flanking regions of the target gene were amplified by PCR using specific primers (Table S3), joined following the Gibson Assembly Master Mix Protocol, and cloned into the pKNG101 vector. The resulting constructs were transferred to *C. violaceum* by triparental conjugation. Transconjugants were selected on LB agar plates containing ampicillin and streptomycin (for pKNG101) to select the first homologous recombination event. Isolated colonies were grown in LB and plated on LB supplemented with 16% sucrose for counter-selection of the second recombination event. Deletion of the target genes was verified by PCR and DNA sequencing. For the construction of strain used in the co-immunoprecipitation assay (JA05), a hemagglutinin (HA) tag was inserted upstream of the *vgrG*3 stop codon in the *C. violaceum* genome by homologous recombination *via* plasmid pSC3913 and confirmed by PCR.

### Interspecies interbacterial competition

The interbacterial competition assay was performed as previously described (31, 62). Attacker (*C. violaceum*) and target strains were grown overnight at 37 °C on LB agar plates, resuspended in liquid LB, normalized to an OD_600_ of 5 and mixed at a 5:1 ratio (attacker: target). Then, 10 μl of the mixture was spotted onto LB agar plates and incubated for 4 h at 37 °C to allow competition. After this period, cells were recovered in 1 ml of LB, serially diluted, and plated on LB agar containing kanamycin (to select *E. coli* BW25113 *lacA*::kan) or nalidixic acid (to select *P. aeruginosa* PA14) to determine the CFU (colony-forming units) of the target strains.

### Intraspecies bacterial competition

Intraspecies competition assays were performed to assess susceptibility to T6SS effector-mediated attack between sibling *C. violaceum* cells. For this, nalidixic acid-sensitive *C. violaceum* strains (attacker) and nalidixic acid-resistant strains (target) were used. Attacker and target strains were grown overnight at 37 °C on LB agar plates, resuspended in liquid LB, normalized to an OD_600_ of 0.5, and mixed at a 1:1 ratio. Then, 10 μl of the mixture was spotted onto LB agar plates, which were incubated at 37 °C for 7 h to allow competition. After this period, cells were recovered in 1 ml of LB, serially diluted, and plated on LB agar containing nalidixic acid to determine the CFU (colony-forming units) of the target strains.

### Toxicity assay assessed by spot viability, growth curves, and light microscopy

The pBAD18-Kan expression vector (63), either empty or containing inserts to allow the expression of toxins with or without their corresponding immunity proteins, was used to transform *E. coli* MG1655. Transformants were grown overnight at 37 °C in LB liquid medium supplemented with kanamycin and 0.5% d-glucose to repress expression of the cloned genes. Cultures were normalized to an OD_600_ of 1, from which 10-fold serial dilutions were prepared in PBS (phosphate-buffered saline). For spot viability, 5 μl of each dilution was spotted onto LB agar plates containing kanamycin and either 0.2% d-glucose or l-arabinose at concentrations of 0.002% or 0.02%. After drying, the plates were incubated at 37 °C overnight before image acquisition.

For growth curves, overnight cultures grown in LB medium supplemented with kanamycin and 0.5% d-glucose were used to inoculate cultures to a starting OD_600_ of 0.01 in LB medium containing kanamycin and either 0.5% d-glucose to repress gene expression or l-arabinose at 0.002%, 0.02%, or 0.2% to induce expression. Growth was monitored in a 96-well plate using a BioTek Synergy HTX Multimode Reader at 37 °C with vigorous and continuous shaking.

For time-lapse microscopy, *E. coli* MG1655 strains carrying pBAD18-Kan-based constructs directing the expression of *rhsF*-CT or *rhsF*-CT/*rhsFi* were grown overnight in LB medium with kanamycin and 0.5% d-glucose, and then these cultures were diluted in liquid LB to an OD_600_ of 0.05. A 10 μl aliquot was pipetted onto a coverslip coated with solid LB medium supplemented with kanamycin and 0.02% l-arabinose. Images were captured for up to 8 h at 3- to 5-min intervals using a Nikon Biostation IM-Q light microscope, which maintains the samples at 37 °C under controlled humidity conditions.

### Immunoblotting to determine the stability of RhsF-CT variants and the expression of VgrG3-HA

Genes encoding variants of RhsF-CT with an N-terminal 3xFLAG epitope tag, with or without RhsFi, were cloned in pBAD18-Kan (Tables S2 and S3). Overnight cultures grown in LB media supplemented with kanamycin and d-glucose 0.5% were used to inoculate cultures to a starting OD_600_ of 0.02 in LB media supplemented with kanamycin. Cells were grown for 2 h at 37 °C, then l-arabinose was added to a final concentration of 0.2% for a further 2 h of growth. Then, 1 ml of culture was harvested by centrifugation, combined with 4X protein loading buffer (Tris-HCl 200 mM, pH 6.8, EDTA 6.4 mM, glycerol 32%, SDS 6.4% and bromophenol blue 0.007%) at a volume proportional to the culture OD_600_ (100 μl of buffer for OD_600_ = 1) and heated at 100 °C for 10 min. For the detection of the VgrG3-HA, protein samples obtained following the co-immunoprecipitation protocol (see section below) were mixed at a 1:1 volume ratio with 4X protein loading buffer. Proteins were separated by 4 to 20% SDS-PAGE and transferred onto PVDF membrane (Millipore) *via* dry transfer using the iBlot2 system (Thermo Fisher). The membrane was blocked with milk powder 2.5% (Marvel) suspended in PBS + 0.1% Tween-20. Membrane washing was done with PBS + 0.1% Tween-20. 3xFLAG-RhsF-CT was detected using the primary antibody anti-FLAG (Sigma Aldrich, #F3165) at 1:10,000, and an anti-EF-Tu (Hycult biotech, #HM6010) was used to provide a loading control. The VgrG3-HA was detected using an anti-HA monoclonal antibody (Sigma Aldrich, #H6533) at 1:5000. The secondary antibody, HRP-conjugated anti-mouse (BioRad, #170-6516) was used at 1:10,000. For signal detection, the membrane was treated with HRP substrate (Millipore) and imaged using an Azure 600 imaging system.

### Preparation of samples for analysis of the *C. violaceum* supernatant by mass spectrometry

To identify T6SS-secreted effectors, *C. violaceum* wild type and Δ*tssB* strains were grown in seven biological replicates overnight in LB at 37 °C. Cultures were diluted to an OD_600_ of 0.04 in 250 ml of minimal medium and incubated for 8 h until reaching an OD_600_ of 1.0. Cultures were subjected to centrifugation at 5000×*g* for 30 min at 4 °C, and the culture supernatant was carefully collected for another round of centrifugation. This process was repeated four times to minimize cellular contamination. After the fourth centrifugation, 100 ml of culture supernatant was collected and treated with 6.25% TCA (trichloroacetic acid) for 14 h at 4 °C. Precipitated proteins were recovered by centrifugation at 5000×*g* for 30 min at 4 °C, and the resulting pellet was washed five times with 80% cold acetone. Protein samples were then dried under laminar airflow for 30 min.

### Proteomics sample preparation, LC-MS, analysis and post-processing

Precipitated protein samples were resuspended in 50 mM triethyl ammonium bicarbonate (TEAB), pH 8.5, with 5% (w/v) SDS and 1× complete protease inhibitor cocktail (Roche). Samples were sonicated using a UP200St ultrasonic processor (Hielscher) at 90 W, 45 s pulse, 15 s rest, three times. Protein concentration was quantified using Pierce BCA Protein Assay (Thermo Fisher). Samples (30 μg) were then denatured with 5 mM TCEP at 60 °C for 15 min, alkylated with 30 mM iodoacetamide at room temperature (20 °C) for 30 min in the dark, and acidified to a final concentration of 2.5% phosphoric acid. Samples were then diluted eightfold with 90% MeOH 10% TEAB (pH 7.2) and added to the S-trap (Protifi) micro columns. The manufacturer-provided protocol was then followed, with a total of five washes in 90% MeOH 10% TEAB (pH 7.2), and trypsin added at a ratio of 1:10 enzyme:protein and digestion performed for 2 h at 47 °C. After elution, peptides were dried using a vacuum concentrator and stored at −80 °C.

Immediately before mass spectrometry, samples were resuspended in 2% acetonitrile 0.1% trifluoroacetic acid in LC-MS grade H_2_O, and each sample was independently analysed on an Orbitrap Exploris 480 mass spectrometer (E480, Thermo Fisher), connected to an UltiMate 3000 RSLCnano System (Thermo Fisher). Peptides (1 μg) were injected on a PepMap 100 C18 LC trap column (300 μm ID × 5 mm, 5 μm, 100 Å) followed by separation on an EASY-Spray nanoLC C18 column (75 μm ID × 50 cm, 2 μm, 100 Å). Solvent A was water containing 0.1% formic acid, and solvent B was 80% acetonitrile containing 0.1% formic acid. The gradient used for analysis of proteome samples was as follows: solvent B was increased from 3 to 5% for 5 min, then from 5 to 35% for 60 min, then increased to 90% for 2.5 min, held at 95% for 2.5 min, then decreased to 3% B in 0.5 min and held at 3% B for 5 min. Flow was kept constant at 400 nl/min. The E480 was operated in positive mode DIA MS2. A precursor ion scan (full scan) was performed in the range 350 to 1050 m/z, 60,000 resolution, normalized AGC target of 150%, RF lens set to 50%. MS2 scans were performed at 30,000 resolution, with isolation windows of 2 m/z, HCD normalized collision energy set to 30%, time to 50 ms. Overall cycletime was 3 s.

Raw files were searched using DIA-NN V 1.8 (64), using its *in silico* generated spectral library function, based on reference proteome FASTA files for *C. violaceum* (UP000001424, downloaded from UniProt on 21/07/2023) and a common contaminants list (65). Trypsin specificity with a maximum of 1 missed cleavage was permitted per peptide, cysteine carbamidomethylation was set as a fixed modification, methionine oxidation as a variable, maximum variable modifications was set to 1. All other variables were left as defaults; protein and peptide false discovery rate (FDR) were set to 1%. Raw data and DIA-NN results files were uploaded to the proteomeXchange *via* the PRIDE partner repository, under PXD071363. Post-processing was performed in R: The DIA-NN output pg.matrix was processed to include only proteins identified by at least 2 peptides, and contaminants were auto-excluded. Protein log2 intensity values were subjected to processing with limma (66), with significance inferred by FDR corrected *t* test (*p* = <0.05) and fold change of ±2-fold.

### Immunoprecipitation of VgrG3-HA and mass spectrometry analysis of associated proteins

Co-immunoprecipitation followed by mass spectrometry (Co-IP/MS), was conducted in biological triplicates. Overnight cultures of wild-type *C. violaceum* (no HA tag, negative control) and *vgrG*3-HA strains were used to inoculate cultures to a starting OD_600_ of 0.05 in 50 ml of LB medium and grown at 37 °C until reaching an OD_600_ of 2.5. Cells were harvested by centrifugation at 48,000×*g* for 20 min at 4 °C, and resuspended in 5 ml of lysis buffer (20 mM Tris-Cl, pH 7.5, 150 mM NaCl, 0.5 mM EDTA, 0.1% Triton X-100, and protease inhibitor cocktail (Roche). Cell lysis was performed by sonication, and the soluble fraction was obtained by centrifugation at 21,000×*g* for 20 min at 4 °C. For Co-IP of proteins using magnetic beads, 30 μl of HA-tag Rabbit mAb magnetic beads (NEB) were washed three times with wash buffer (20 mM Tris-Cl pH 7.5, 150 mM NaCl, 0.5 mM EDTA, 0.1% Triton X-100) and incubated with the entire soluble fraction for 16 h at 4 °C under gentle rotation. After incubation, the supernatant was discarded, and the beads were washed three times. Protein elution was performed using 30 μl of elution buffer (5% SDS, 50 mM Tris, pH 7.5, 150 mM NaCl), incubated at 70 °C for 5 min.

Sample preparation and trypsin digestion for mass spectrometry were performed at the Proteomics Facility of the Centre for Advanced Scientific Technologies, University of Dundee. Protein concentrations were determined using the Micro-BCA assay (Thermo Scientific) and were processed using S-Trap Micro columns (Protifi) where proteins were reduced, alkylated and digested overnight at 37 °C at 1:40 enzyme-to-substrate. A second digest was repeated for 6 h the following day. Digested peptides were run on a Q-Exactive Plus (Thermo Scientific) instrument coupled to a Dionex Ultimate 3000 HPLC system (Thermo Scientific) with LC buffers comprising buffer A (0.1% formic acid) and buffer B (80% acetonitrile, 0.1% formic acid). The buffers were used to create a gradient lasting 185 min where the peptides were eluted from a 200 cm uPAC nanoLC C18 column (Pharma Fluidics) at a flow rate of 300 nl/min for the first 156 min before increasing to 400 nl/min for the remainder of the gradient. Raw data were acquired in Data Independent Acquisition (DIA) mode. A scan cycle compromised a full MS scan with an m/z range of 345 to 1155, resolution of 70,000, Automatic Gain Control (AGC) target 3 × 10^6^ and a maximum injection time of 200 ms. MS scans were followed by DIA scans of dynamic window widths with an overlap of 0.5 Th. DIA spectra were recorded with a first fixed mass of 200 m/z, resolution of 17,500, AGC target 3 × 10^6^ and a maximum IT of 55 ms. Normalized collision energy was set to 25% with a default charge state set at 3. Data for both MS scan and MS/MS DIA scan events were acquired in profile mode. Peptides were initially trapped on an Acclaim PepMap C18 (100 μm × 2 cm) and then separated on a 200 cm uPAC nanoLC C18 column (Pharma Fluidics). The column was kept at a constant temperature of 50 ˚C and a source voltage of 2.0 kV. Data are available *via* ProteomeXchange with identifier PXD071369. Label-free analysis was performed in MaxQuant version 1.6.2.10 using the RAW files. Further data analysis was performed using Perseus version 1.6.12.0 to generate *p*-values and fold change and perform Student t-tests.

### Cloning, expression, and purification of the RhsF-CT/RhsFi complex

The RhsF-CT/RhsFi complex was expressed using the pACYCDuet-1 expression vector, which contains two multiple cloning sites (MCSs) and is designed for the co-expression of two genes. The DNA fragment encoding the full-length RhsFi immunity protein was cloned into MCS-2. Subsequently, the DNA fragment encoding the toxic C-terminal portion of RhsF (RhsF-CT, starting from amino acid 1393 of RhsF) plus His_6_Tag and TEV protease recognition sequence was amplified from pET15b-TEV (67) and cloned into MCS-1. For protein production, *E. coli* BL21(DE3) was freshly transformed with pSC3912, and the transformed cells were grown in liquid LB medium supplemented with chloramphenicol at 30 °C until reaching an OD_600_ of 0.8. The temperature was then reduced to 20 °C, and expression was induced overnight with 1 mM isopropyl β-D-1-thiogalactopyranoside (IPTG). Cells were harvested by centrifugation and resuspended in lysis buffer (50 mM Tris-HCl pH 8.0, 250 mM NaCl, 30 mM imidazole, and 0.5 mM TCEP), supplemented with DNaseI (Sigma Aldrich) and an EDTA-free protease inhibitor cocktail (Sigma Aldrich). Cell lysis was performed using a cell disruptor at 30 pounds per square inch (PSI). The soluble fraction was obtained by centrifugation at 40,000 *g* for 30 min at 4 °C and clarified with 0.22 μm filters.

The His_6_-RhsF-CT/RhsFi complex was purified using a 5 ml Cytiva Ni^2+^ HisTrap HP column (GE Healthcare). After loading the soluble fraction, the column was washed with lysis buffer, and the complex was eluted using linear gradient of imidazole. Desalting was then performed using a HiPrep 23/10 Desalting column into storage buffer (50 mM Tris-HCl pH 8.0, 250 mM NaCl, 0.5 mM TCEP). For X-ray crystallography, the His_6_-tag was removed by treating purified complex with TEV protease (1 mg of TEV per 10 mg of protein) overnight at 4 °C. To eliminate any remaining His_6_-tagged protein, cleaved tag and TEV-protease, the TEV-digested sample was subjected to reverse purification on a HisTrap HP column (GE Healthcare). Finally, the purified complex was subjected to size-exclusion chromatography (SEC) on Superdex 75 HiLoad 16/600 column (GE Healthcare) equilibrated with buffer (20 mM Tris-HCl pH 8.0, 150 mM NaCl, and 0.5 mM TCEP-HCl). The proteins were loaded with either 1.5 ml or 2 ml loops and eluted with the same buffer. For the ADP-ribosylation assays, the RhsF-CT was purified from the His_6_-RhsF-CT/RhsI complex into a Ni^2+^ column following a previously described guanidine/urea-based unfolding and refolding protocol (53).

### X-ray crystallography and diffraction data collection

Crystallization condition screening for the RhsF-CT/RhsFi complex was performed using commercial 96-well screening plates from Molecular Dimensions Ltd and Qiagen. The sitting-drop vapor diffusion method was used, combining 0.1 μl of protein solution (25 mg/ml) with 0.1 or 0.2 μl of reservoir solution. Crystallization plates were incubated at 20 °C, and crystal formation was monitored under a polarized light stereomicroscope. The four crystals selected for X-ray diffraction were obtained from the PACT Premier screen (Molecular Dimensions), after supplementing the RhsF-CT/RhsFi purification buffer with 20% ethanol. These crystals were obtained under the following specific conditions: Complex concentration: 8.33 mg/ml, pH 6.0; Ethanol 6.7%; PEG1500 16.7%; Tris-Cl 16.7 mM; NaCl 83.3 mM; and MMT buffer (malic acid, MES, and Tris) 67 mM.

X-ray diffraction data were collected and processed at the Diamond Light Source (DLS) synchrotron facility in Didcot, UK, using standard protocols. Reservoir solutions supplemented with 20 to 25% (v/v) glycerol were used as cryoprotectants for the four selected RhsF-CT/RhsFi crystals. Individual crystals were frozen, and diffraction data were collected at beamline I03 using an Eiger2 XE 16M detector. Crystals were maintained at 100 K using a gaseous nitrogen stream. For each crystal, 360 diffraction images were collected with an oscillation of 0.1° per image. The crystal-to-detector distances were 200 mm (for Crystals 1 and 3) and 290 mm (for Crystals 2 and 4), using an X-ray wavelength of 0.9763 Å. The collected diffraction data were indexed, integrated, and scaled using XDSgui (68). The Matthews coefficients (Å3.Da-1) and solvent content (%) were calculated using Xtriage within the Phenix software suite (69).

For structure refinement, the top-ranked ColabFold (70) model of the RhsF-CT/RhsFi complex was iteratively refined and manually adjusted using Phenix (69) and Coot (71), respectively. After initial refinement cycles, water molecules were added automatically by Phenix and manually verified based on electron density shape and hydrogen bonding potential. Throughout refinement, model geometry, structural quality, and fit to experimental data were monitored in Coot and evaluated using key parameters such as R_free_, R_work_, clashscore, RMSD (bond lengths and angles), and B-factors *via* MolProbity (72) and other Phenix tools (*e.g.*, POLYGON) (73). This refinement procedure was performed separately for all four datasets, with progressive reduction in R_free_ values. The dataset yielding the highest data completeness and lowest values for resolution, R_free_, R_work_, and clashscore, alongside angle RMSD close to 1 and bond RMSD near 0.01, was selected as the final model. Structural figures were generated using Chimera and ChimeraX (74).

### ADP-ribosylation assay and immunoblotting detection of ADP-ribosylated products

*In vitro* ADP-ribosylation reactions were performed in a total volume of 20 μl containing 50 mM Bis-Tris pH 6.5, 300 mM NaCl, 0.05 μM NAD^+^ (Sigma-Aldrich #N1511), 0.5 μM of purified RhsF-CT or RhsF-CT/RhsFi complex, and 10 μM of a 25-bp RNA primer (Table S3). Full reactions or control reactions lacking either protein or NAD^+^ were incubated at 37 °C for 1 h. Following incubation, samples were resolved by electrophoresis on 15% denaturing TBE-urea polyacrylamide gels (Biorad #3450091). After RNA visualization by SYBR Gold staining (Thermo Fisher #S11494), the separated products were transferred to a positively charged nylon membrane (BrightStar), using the same transfer buffer as for standard immunoblotting, applying 400 mA for 35 min in cold buffer. Following transfer, RNA was crosslinked to the membrane by UV irradiation for 1 min. Membranes were then blocked with 0.2% (w/v) I-Block (Invitrogen) in PBS containing 0.05% Tween-20. Immunoblot detection was performed by incubating the membrane overnight at 4 °C with an anti-poly/mono-ADP ribose antibody (Cell Signalling #71630SF) at 1:5000, and a secondary anti-rabbit peroxidase-conjugated antibody (Biorad #70-6515) at 1:10.000.

## Data availability

The crystal structure of the RhsF-CT/RhsFi complex has been deposited into the Protein Data Bank (PDB) under the accession code 9ZDM. The mass spectrometry proteomics data are available in the ProteomeXchange Consortium *via* the PRIDE partner repository with the dataset identifier PXD071363 for the secretome analysis and PXD071369 for the co-immunoprecipitation of VgrG3. All other data are included within the manuscript or the supporting information.

## Supporting information

This article contains supporting information (five Supporting Figures and three Supporting Tables). (61, 63, 67)

## Conflict of interest

The authors declare that they have no conflicts of interest with the contents of this article.
