# Supplementary material for: Global identification of Chromobacterium violaceum T6SS effectors reveals an Rhs antibacterial toxin featuring FIX and ADP-ribosyltransferase domains
Source: J Biol Chem. 2026 Jun 1;302(7):113216. doi: 10.1016/j.jbc.2026.113216 (PMC13324451; doi:10.1016/j.jbc.2026.113216)
Supplement: Supporting Tables and Figures [file mmc1.pdf]

Global identification of *Chromobacterium violaceum* T6SS effectors reveals an Rhs antibacterial toxin featuring FIX and ADP-ribosyltransferase domains

Júlia A Alves[1,2], Genady Pankov[2], Andrew Frey[3], Matthias Trost[3], Germán G Sgro[4], Sarah J Coulthurst[2,*], and José F da Silva Neto[1,*]

Figure S1. Strategies for identification of *C. violaceum* T6SS effectors.

Figure S2. **Time-lapse microscopy of *E. coli* expressing RhsF or RhsF/RhsFi.**

Figure S3. **Crystallization of the RhsF-CT/RhsFi complex.**

Figure S4. Residues involved in the protein interface between RhsF-CT and RhsFi.

Figure S5: Toxicity assays of the FLAG-tagged RhsF-CT variants.

Table S1. List of proteins showing structural similarity with RhsF-CT.

Table S2. Plasmids used in this work.

Table S3. Oligonucleotide primers and synthetic gene fragments.

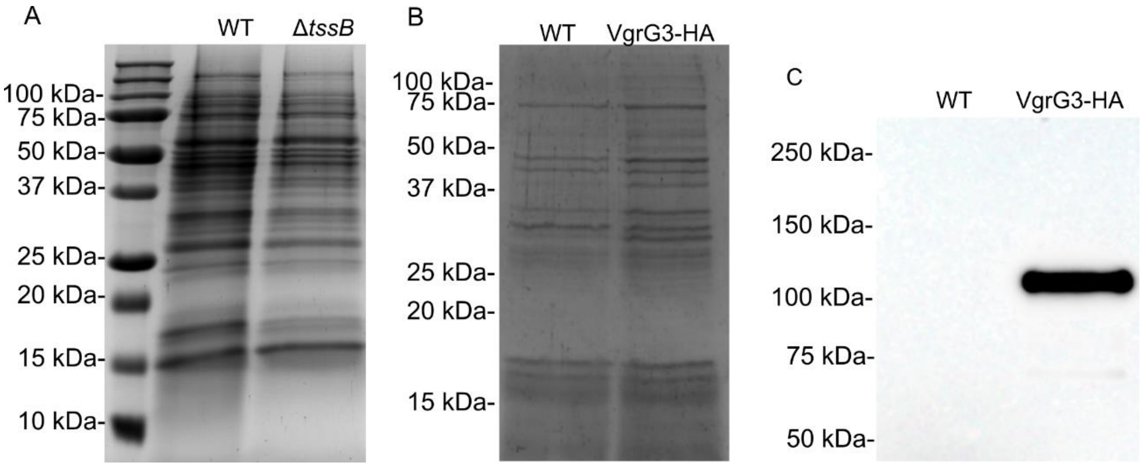

Figure S1. Strategies for identification of *C. violaceum* T6SS effectors. *A,* proteins secreted by *C. violaceum* wild-type (WT) and Δ*tssB* strains, obtained by TCA precipitation and resolved by SDS-PAGE. *B,* proteins eluted from an anti-HA immunoprecipitation performed on cell lysates of wild type *C. violaceum* (negative control) or the strain encoding VgrG3-HA, resolved by SDS-PAGE and stained with Coomassie Blue. *C,* anti-HA immunoblotting, confirming the expression of the VgrG3-HA fusion protein (107 kDa).

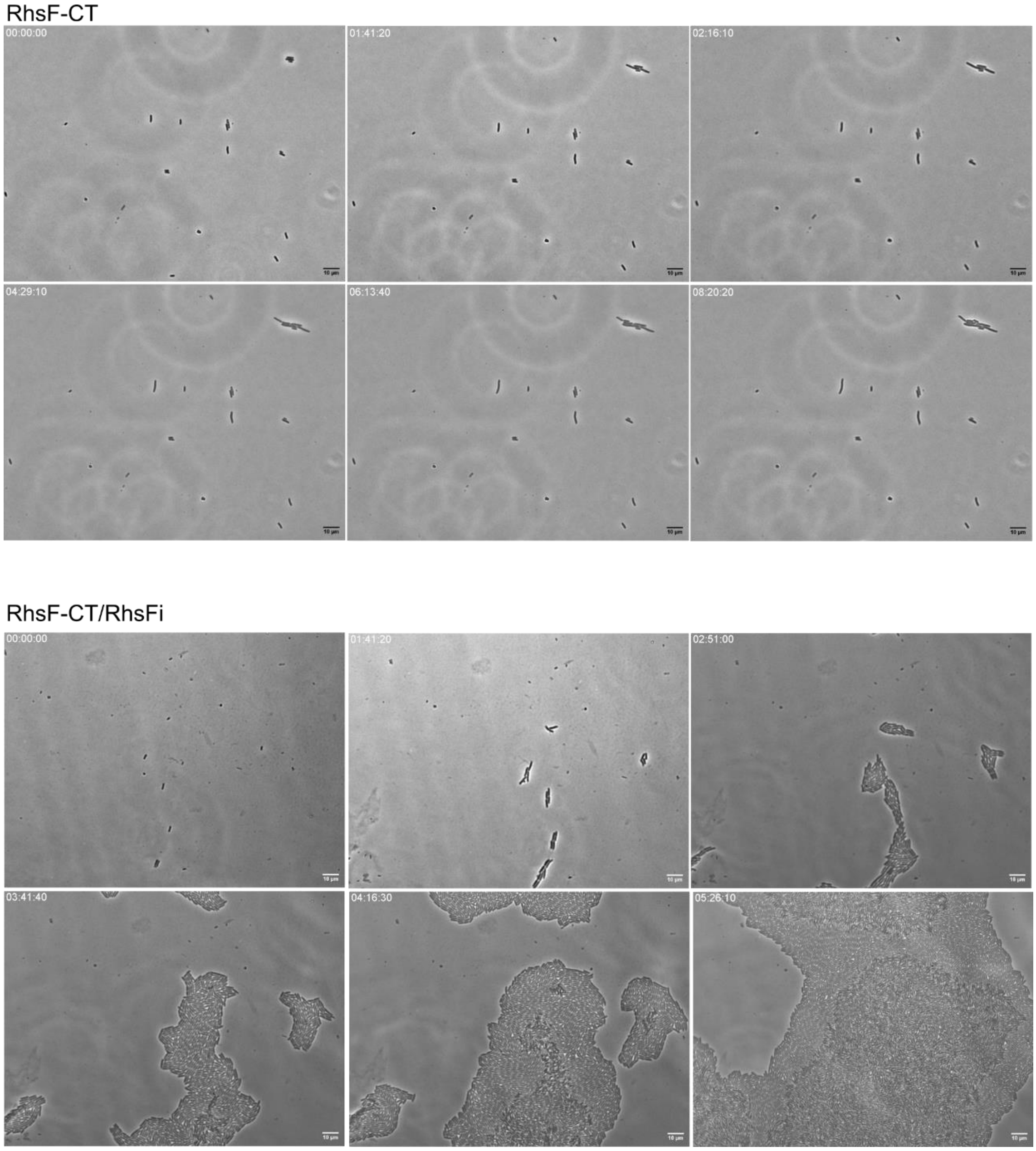

Figure S2. **Time-lapse microscopy of *E. coli* expressing RhsF or RhsF/RhsFi.** Light microscopy of *E. coli* MG1655 carrying the pBAD18-Kan vector expressing the toxic C-terminal domain of RhsF (RhsF-CT) or co-expressing RhsF-CT with the immunity protein RhsFi. Strains were monitored for approximately 8 hours to evaluate their growth.

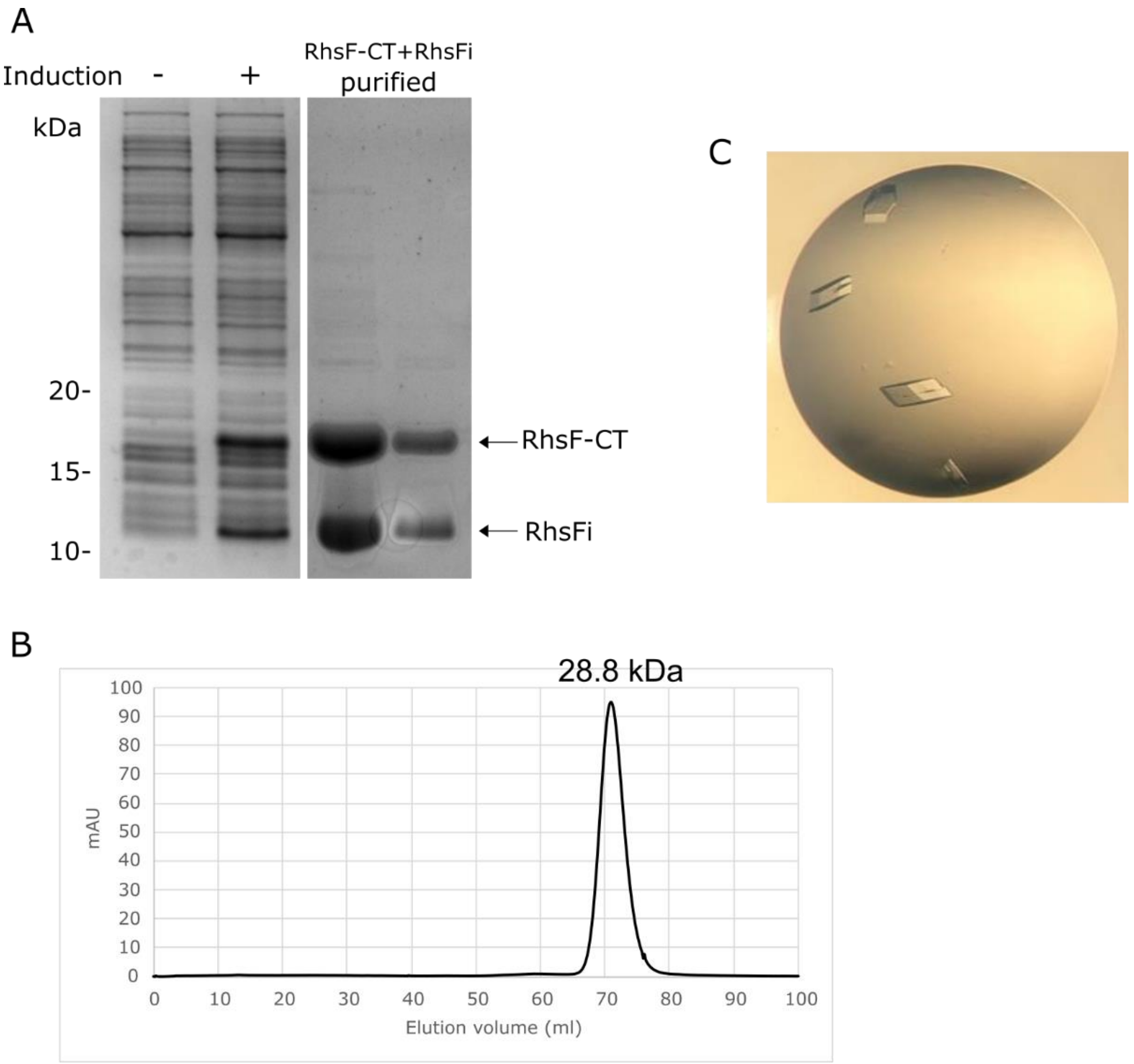

Figure S3. **Crystallization of the RhsF-CT/RhsFi complex.** *A,* SDS-PAGE analysis of total protein with (+) or without (-) induction of production of the RhsF-CT and RhsFi proteins by addition of IPTG to cultures of *E. coli* BL21(DE3) carrying pSC3912 (left), and of eluted proteins following nickel affinity purification of the RhsF-CT/RhsFi complex (right). *B,* size exclusion chromatography on Superdex 75 HiLoad 16/600 column (GE Healthcare) of the purified RhsF-CT/RhsFi complex. The elution volume corresponds to a complex with 28.8 kDa. *C,* image of the four RhsF-CT/RhsFi crystals obtained and submitted for X-ray diffraction analysis.

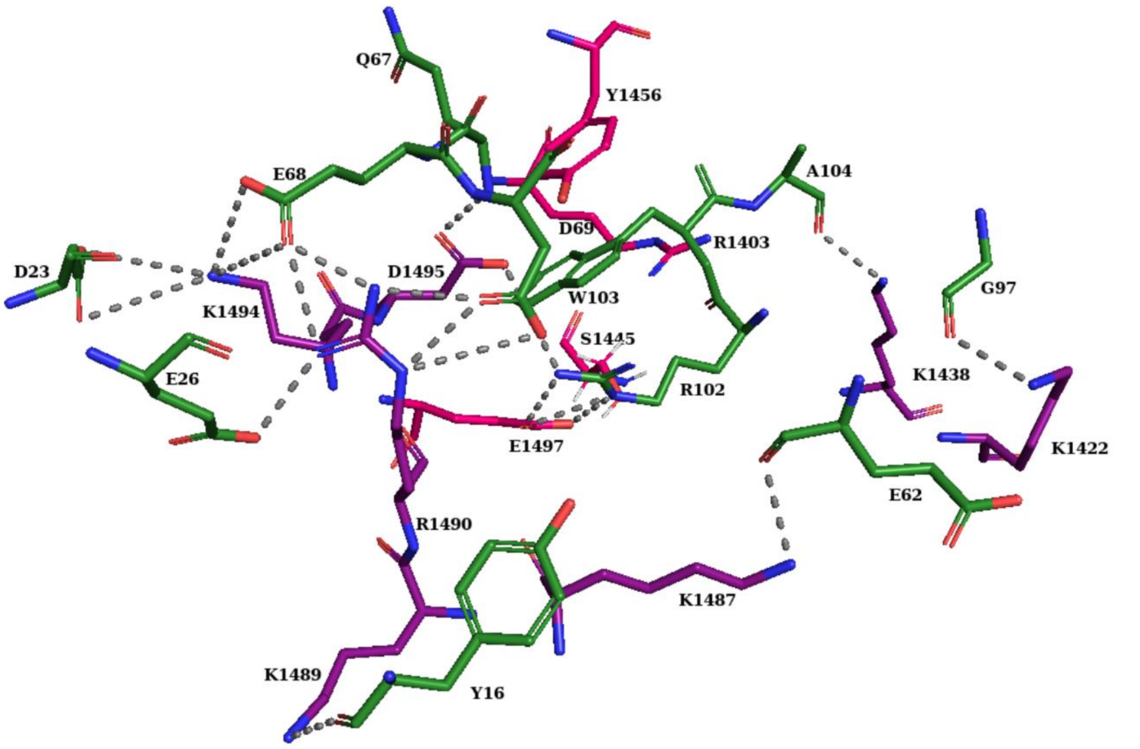

Figure S4. Residues involved in the protein interface between RhsF-CT and RhsFi. Carbon atoms are coloured purple for RhsF-CT and green for RhsFi, while the carbon atoms of residues E1497, S1445, Y1456, and R1403 in RhsF-CT are highlighted in pink. Salt bridges and hydrogen bonds are indicated by dashed lines. These were identified using PDBePISA (https://www.ebi.ac.uk/pdbe/pisa/) based on the crystallographic structure. Salt bridges were defined and counted per N–O atom pair with distance ≤ 4.0 Å between oppositely charged side chain.

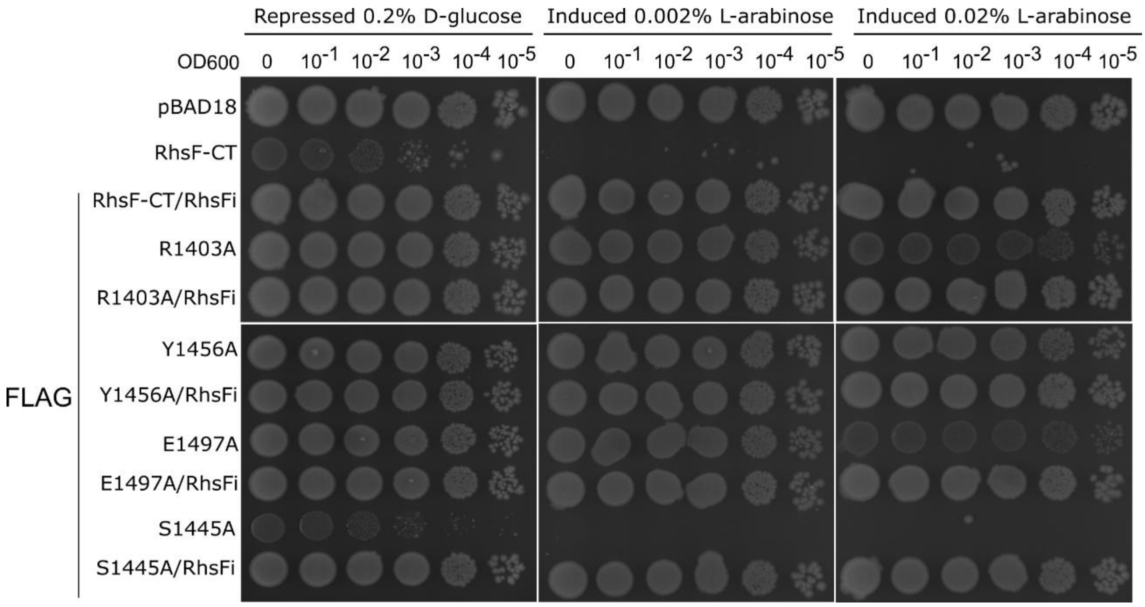

Figure S5: Toxicity assays of the FLAG-tagged RhsF-CT variants. Growth of *E. coli* MG1655 carrying pBAD18-Kan (vector control) or derivatives directing the expression of the C-terminal toxin domain of RhsF (RhsF-CT) or its FLAG-tagged variants, alone or co-expressed with RhsFi, on LB media containing either D-glucose (repression) or L-arabinose (induction) for regulation of gene expression.

Table S1. Proteins showing structural similarity with RhsF-CT.

PDB entry	Z-score	Q-score	RMSD	Sequence identity %	Target (PDB entry)	Protein name	Organism
5zj4:A	7.4	0.34	1.96	22	Guanine-specific ADP ribosyltransferase	ScARP	*Streptomyces coelicolor*
5zj5:B	7.3	0.30	1.98	23	Guanine-specific ADP ribosyltransferase with NADH and GDP	ScARP	*Streptomyces coelicolor*
4z9d:A	8.4	0.28	2.15	17	EcPltA	EcPltA	*Escherichia coli*

Proteins identified using PDB e-Fold with RMSD (root mean square deviation) ≤ 2.5 and Z-score (standard score) ≥ 5.0 are included.

Table S2. Plasmids used in this work.

**Plasmids**	**Description**	**Source/Reference**
pBAD18-Kan	Arabinose-inducible expression vector; gene of interest is cloned downstream of the Para promoter (KanR)	(Guzman et al., 2000)
pACYC-Duet-1	Protein overproduction vector for the co-expression of two genes. Each multiple cloning site (MCS) is preceded by a T7 promoter, and the first site allows for fusion of an N-terminal His6 tag (KanR)	Novagen
pKNG101	Suicide vector for allelic exchange (SmR, sacBR, mobRK2, oriR6K)	(Kaniga et al., 1991)
pET15b-TEV	Vector for protein overexpression under the control of the T7 promoter. Permits fusion of an His6 tag followed by a TEV protease cleavage site to the N-terminus of the overexpressed protein (ApR)	(Rao et al., 2011)
pSC3904	pKNG101-derived allelic exchange plasmid for generation of an in-frame deletion of *rhsF* (CV_1431) and *rhsFi* (CV_1430)	This work
pSC3905	pKNG101-derived allelic exchange plasmid for generation of an in-frame deletion of *rhsF*	This work
pSC3913	pKNG101-derived allelic exchange plasmid for the genomic insertion of a hemagglutinin epitope at the C-terminal region of VgrG3 (CV1432)	This work
pSC3911	Coding sequences for RhsF-CT and RhsFi in pET15b-TEV. RhsF fused to His6Tag and TEV sequence	This work
pSC3912	Coding sequences for RhsF-CT and RhsFi in pACYC-Duet. *rhsF* fused with TEV and His6Tag was amplifyed from pSC3911 and cloned into the first MCS	This work
pSC3948	Coding sequence for 3xFLAG-RhsF-CT (CV_1431; amino acids 1393- 1513) and RhsFi (CV_1430) in pBAD18-Kan. Produced by Genscript	This work
pSC3949	Coding sequence for 3xFLAG-RhsF-CT R1403A and RhsFi (CV_1430) in pBAD18-Kan. Produced by Genscript	This work
pSC3950	Coding sequence for 3xFLAG-RhsF-CT Y1456A and RhsFi (CV_1430) in pBAD18-Kan. Produced by Genscript	This work
pSC3951	Coding sequence for 3xFLAG-RhsF-CT E1497A and RhsFi (CV_1430) in pBAD18-Kan. Produced by Genscript	This work
pSC3967	Coding sequence for 3xFLAG-RhsF-CT S1445A and RhsFi (CV_1430) in pBAD18-Kan. Produced by Genscript	This work
pSC3952	Coding sequence for 3xFLAG-RhsF-CT in pBAD18-Kan. Derived from pSC3948 by digestion with *SalI*	This work
pSC3953	Coding sequence for 3xFLAG-RhsF-CT R1403A in pBAD18-Kan. Derived from pSC3949 by digestion with *SalI*	This work
pSC3954	Coding sequence for 3xFLAG-RhsF-CT Y1456A in pBAD18-Kan. Derived from pSC3950 by digestion with *SalI*	This work

pSC3955	Coding sequence for 3xFLAG-RhsF-CT E1497A in pBAD18-Kan. Derived from pSC3951 by digestion with *SalI*	This work
pSC3969	Coding sequence for 3xFLAG-RhsF-CT S1445A in pBAD18-Kan. Derived from pSC3967 by digestion with *SalI*.	This work

Table S3. Oligonucleotide primers and synthetic gene fragments.

Plasmid	Sequence 5'- 3'	Description
	AUGCAGUGGAAGUGAUGACGUGAU	RNA primer used as probe in ADP-ribosylation assays
pSC3904	tcccccccccccctgcaggtcgacCAGGGCAAGTTCGTCGTC	Forward primer to clone upstream region of *rhsF* and *rhsFi* in pKNG101 (Made with Gibson assembly)
	taaccaagtcCGCTTTGGAAGCATTGGC	Reverse primer to clone upstream region of *rhsF* and *rhsFi* in pKNG101 (Made with Gibson assembly)
	ttccaaagcgGACTTGGTTATAGGCTCGCC	Forward primer to clone downstream region of *rhsF* and *rhsFi* in pKNG101 (Made with Gibson assembly)
	tccacggactatagactatactagtGCCAAGCGGGTCGTAGTG	Reverse primer to clone downstream region of *rhsF* and *rhsFi* in pKNG101 (Made with Gibson assembly)
pSC3905	tcccccccccccctgcaggtcgacCAGGGCAAGTTCGTCGTC	Forward primer to clone upstream region of *rhsF* in pKNG101 (Made with Gibson assembly)
	taacctcgtaCGCTTTGGAAGCATTGGC	Reverse primer to clone upstream region of *rhsF* in pKNG101 (Made with Gibson assembly)
	ttccaaagcgTACGAGGTTATAGAGTAAGGAGTATTTTTATGAG	Forward primer to clone downstream region of *rhsF* in pKNG101 (Made with Gibson assembly)
	tccacggactatagactatactagtATGCCAGGGTCTGCTTGG	Reverse primer to clone downstream region of *rhsF* in pKNG101 (Made with Gibson assembly)
pSC3911	GATCAT CTCGAG ACGGGGAAACAGTTCACAGGG	Forward primer to clone sequence of CT domain of *rhsF* and *rhsFi* in pET15-TEV for protein purification (XhoI)
	GATCAT GGATCC CTAAGCCCATCTAGATAATG	Reverse primer to clone sequence of CT domain of *rhsF* and *rhsFr* in pET15-TEV for protein purification (XhoI)

pSC3912	GATCAT CCATGG GCAGCAGCCATCATCATCATC	Forward primer to amplify His6Tag-TEV-*rhsF* from pSC3911 (*NcoI*)
	GATCAT GGATCC TTACTCTATAACCTCGTACGAAC	Reverse primer to amplify His6Tag-TEV-*rhsF* from pSC3911 (*BamHI*)
	GATCAT CATATG AGTGAACGACTTGAGGCGATA	Forward primer to amplify *rhsFi* to clone into the second MCS (*NdeI*)
	GATCAT GGTACC CTAAGCCCATCTAGATAATGGC	Reverse primer to amplify *rhsFi* to clone into the second MCS (*KpnI*)
pSC3913	GGATCCCCCGGGCTGCAG	Forward primer to amplify pBluescript for subcloning of insert into pKNG101 (*BamHI*)
	TCTAGAGCGGCCGCCACC	Reverse primer to amplify pBluescript for subcloning of insert into pKNG101 (*XbaI*)
	gcggtggcggccgctctagaGTGCTGCTCACCAGCGGC	Forward primer to amplify *vgrG3* fused to HA at C-terminal domain. Insert 1 for Gibson assembly (*XbaI*)
	*agcgtaatctggaacatcgtatgggta*GAACAGTTTGGGCAGGCTGG	Reverse primer to amplify *vgrG3* fused to HA at C-terminal domain. Insert 1 for Gibson assembly
	*tacccatacgatgttccagattacgct*TGACATCACAAAATTAAAATCCATAAAGTTGAATTGCCATG	Forward primer to amplify *vgrG3* fused to HA at C-terminal domain. Insert 2 for Gibson assembly
	tcctgcagcccgggggatccCTGTGCCAGTGCTGCGCA	Reverse primer to amplify *vgrG3* fused to HA at C-terminal domain. Insert 2 for Gibson assembly (*BamHI*)
**Plasmid**	**Details of synthetic insert**	
pSC3948	Synthetic insert containing coding sequences for RhsF-CT tagged with 3×FLAG at the N-terminus, and RhsFi.	
	CAGAGGAATACATATATGGACTACAAAGACCATGACGGTGATTATAAAGATCATGATATCGAT<br>TACAAGGATGACGATGACAAAACGGGGAAACAGTTCACAGGGACGGTTTACCGGGCACTTACA<br>CCGAAGCAAAAGCAATGTGCATTAAAAAATCAAGATATATCCCCAAAGGACCCTAGCGCTAGT<br>TATAGCCCTCAGGAACATGTGGAAAATGGAAAACTGCAAACACAGTTTATTTCAACAACTAAA<br>AGTGAAAAAACAAGCGACTTTTATAATAAATGCAATTGTAAAATAAAAGTAGATCTGTCAAAG<br>ATACCTGATTGCGACATCATTGATGTTAGCAAGGGCCAAGGATTGACAGGAAAAGCTAAGCGG<br>TTTGCCACTAAAGACCAAGAAGTATTAATAAGAAATGGCATTCCTAAGGGTTCGTACGAGGTT<br>ATAGAGTAAGTCGACGTAAGGAGTATTTTTATGAGTGAACGACTTGAGGCGATAAAAAAGAAT<br>TTAGATGATTTTTATGATAAATCTTTTGATTCGGATGATATAGAGAGAGCTGAAAATAAAAGTA<br>TCAAAGAAGAGATAGTGGACCTGATAATTCATGCACATAAAAATAGGGATTATCAGCTTGTGA<br>AGGAAAGTATTGATGTTTTGATTGAAAACACAGGATGCCAAGAGGATTTTGAAATTCTTGAAG<br>AAATAGTTTCTCCTCTTCAATCTGCTGGGATTTTGTCGGACCCTGAACTGAATGACTTGGTTATA<br>GGCTCGCCATTATCTAGATGGGCTTAGGTCGAC	

<table>
<tr><td rowspan="2">pSC3949</td><td>Synthetic insert containing coding sequences for RhsF-CT R1403A with 3×FLAG at the N-terminus, and RhsFi.</td></tr>
<tr><td>CAGAGGAATACATATATGGACTACAAAGACCATGACGGTGATTATAAAGATCATGATATCGAT
TACAAGGATGACGATGACAAAACGGGGAAACAGTTCACAGGGACGGTTTACGCTGCACTTACA
CCGAAGCAAAAGCAATGTGCATTAAAAAATCAAGATATATCCCCAAAGGACCCTAGCGCTAGT
TATAGCCCTCAGGAACATGTGGAAAATGGAAAACTGCAAACACAGTTTATTTCAACAACTAAA
AGTGAAAAAACAAGCGACTTTTATAATAAATGCAATTGTAAAATAAAAGTAGATCTGTCAAAG
ATACCTGATTGCGACATCATTGATGTTAGCAAGGGCCAAGGATTGACAGGAAAAGCTAAGCGG
TTTGCCACTAAAGACCAAGAAGTATTAATAAGAAATGGCATTCCTAAGGGTTCGTACGAGGTT
ATAGAGTAAGTCGACGTAAGGAGTATTTTTATGAGTGAACGACTTGAGGCGATAAAAAAGAAT
TTAGATGATTTTTATGATAAATCTTTTGATTCGGATGATATAGAGAGAGCTGAAAATAAAAGTA
TCAAAGAAGAGATAGTGGACCTGATAATTCATGCACATAAAAATAGGGATTATCAGCTTGTGA
AGGAAAGTATTGATGTTTTGATTGAAAACACAGGATGCCAAGAGGATTTTGAAATTCTTGAAG
AAATAGTTTCTCCTCTTCAATCTGCTGGGATTTTGTCGGACCCTGAACTGAATGACTTGGTTATA
GGCTCGCCATTATCTAGATGGGCTTAGGTCGA</td></tr>
<tr><td rowspan="2">pSC3950</td><td>Synthetic insert containing coding sequences for RhsF-CT Y1456A with 3×FLAG at the N-terminus, and RhsFi.</td></tr>
<tr><td>CAGAGGAATACATATATGGACTACAAAGACCATGACGGTGATTATAAAGATCATGATATCGAT
TACAAGGATGACGATGACAAAACGGGGAAACAGTTCACAGGGACGGTTTACCGGGCACTTACA
CCGAAGCAAAAGCAATGTGCATTAAAAAATCAAGATATATCCCCAAAGGACCCTAGCGCTAGT
TATAGCCCTCAGGAACATGTGGAAAATGGAAAACTGCAAACACAGTTTATTTCAACAACTAAA
AGTGAAAAAACAAGCGACTTTgcgAATAAATGCAATTGTAAAATAAAAGTAGATCTGTCAAAGA
TACCTGATTGCGACATCATTGATGTTAGCAAGGGCCAAGGATTGACAGGAAAAGCTAAGCGGT
TTGCCACTAAAGACCAAGAAGTATTAATAAGAAATGGCATTCCTAAGGGTTCGTACGAGGTTA
TAGAGTAAGTCGACGTAAGGAGTATTTTTATGAGTGAACGACTTGAGGCGATAAAAAAGAATT
TAGATGATTTTTATGATAAATCTTTTGATTCGGATGATATAGAGAGAGCTGAAAATAAAAGTAT
CAAAGAAGAGATAGTGGACCTGATAATTCATGCACATAAAAATAGGGATTATCAGCTTGTGAA
GGAAAGTATTGATGTTTTGATTGAAAACACAGGATGCCAAGAGGATTTTGAAATTCTTGAAGA
AATAGTTTCTCCTCTTCAATCTGCTGGGATTTTGTCGGACCCTGAACTGAATGACTTGGTTATAG
GCTCGCCATTATCTAGATGGGCTTAGGTCGA</td></tr>
<tr><td rowspan="2">pSC3951</td><td>Synthetic insert containing coding sequences for RhsF-CT E1497A with 3×FLAG at the N-terminus, and RhsFi.</td></tr>
<tr><td>CAGAGGAATACATATATGGACTACAAAGACCATGACGGTGATTATAAAGATCATGATATCGAT
TACAAGGATGACGATGACAAAACGGGGAAACAGTTCACAGGGACGGTTTACCGGGCACTTACA
CCGAAGCAAAAGCAATGTGCATTAAAAAATCAAGATATATCCCCAAAGGACCCTAGCGCTAGT
TATAGCCCTCAGGAACATGTGGAAAATGGAAAACTGCAAACACAGTTTATTTCAACAACTAAA
AGTGAAAAAACAAGCGACTTTTATAATAAATGCAATTGTAAAATAAAAGTAGATCTGTCAAAG
ATACCTGATTGCGACATCATTGATGTTAGCAAGGGCCAAGGATTGACAGGAAAAGCTAAGCGG
TTTGCCACTAAAGACCAAgcgGTATTAATAAGAAATGGCATTCCTAAGGGTTCGTACGAGGTTAT
AGAGTAAGTCGACGTAAGGAGTATTTTTATGAGTGAACGACTTGAGGCGATAAAAAAGAATTT
AGATGATTTTTATGATAAATCTTTTGATTCGGATGATATAGAGAGAGCTGAAAATAAAAGTATC
AAAGAAGAGATAGTGGACCTGATAATTCATGCACATAAAAATAGGGATTATCAGCTTGTGAAG
GAAAGTATTGATGTTTTGATTGAAAACACAGGATGCCAAGAGGATTTTGAAATTCTTGAAGAA
ATAGTTTCTCCTCTTCAATCTGCTGGGATTTTGTCGGACCCTGAACTGAATGACTTGGTTATAGG
CTCGCCATTATCTAGATGGGCTTAGGTCGA</td></tr>
<tr><td rowspan="2">pSC3967</td><td>Synthetic insert containing coding sequences for RhsF-CT S1445A tagged with 3×FLAG at the N-terminus, and RhsFi.</td></tr>
<tr><td>GAGCTCAGAGGAATACATATATGGACTACAAAGACCATGACGGTGATTATAAAGATCATGATA
TCGATTACAAGGATGACGATGACAAAACGGGGAAACAGTTCACAGGGACGGTTTACCGGGCAC
TTACACCGAAGCAAAAGCAATGTGCATTAAAAAATCAAGATATATCCCCAAAGGACCCTAGCG
CTAGTTATAGCCCTCAGGAACATGTGGAAAATGGAAAACTGCAAACACAGTTTATTGCTACAA
CTAAAAGTGAAAAAACAAGCGACTTTTATAATAAATGCAATTGTAAAATAAAAGTAGATCTGT
CAAAGATACCTGATTGCGACATCATTGATGTTAGCAAGGGCCAAGGATTGACAGGAAAAGCTA
AGCGGTTTGCCACTAAAGACCAAGAAGTATTAATAAGAAATGGCATTCCTAAGGGTTCGTACG
AGGTTATAGAGTAAGTCGACGTAAGGAGTATTTTTATGAGTGAACGACTTGAGGCGATAAAAA
AGAATTTAGATGATTTTTATGATAAATCTTTTGATTCGGATGATATAGAGAGAGCTGAAAATAA
AAGTATCAAAGAAGAGATAGTGGACCTGATAATTCATGCACATAAAAATAGGGATTATCAGCT
TGTGAAGGAAAGTATTGATGTTTTGATTGAAAACACAGGATGCCAAGAGGATTTTGAAATTCTT
GAAGAAATAGTTTCTCCTCTTCAATCTGCTGGGATTTTGTCGGACCCTGAACTGAATGACTTGG
TTATAGGCTCGCCATTATCTAGATGGGCTTAGGTCGACCTGCAG</td></tr>
</table>
